# Automatic detection for bioacoustic research: a practical guide from and for biologists and computer scientists

**DOI:** 10.1111/brv.13155

**Published:** 2024-10-17

**Authors:** Arik Kershenbaum, Çağlar Akçay, Lakshmi Babu‐Saheer, Alex Barnhill, Paul Best, Jules Cauzinille, Dena Clink, Angela Dassow, Emmanuel Dufourq, Jonathan Growcott, Andrew Markham, Barbara Marti‐Domken, Ricard Marxer, Jen Muir, Sam Reynolds, Holly Root‐Gutteridge, Sougata Sadhukhan, Loretta Schindler, Bethany R. Smith, Dan Stowell, Claudia A.F. Wascher, Jacob C. Dunn

**Affiliations:** ^1^ Girton College and Department of Zoology University of Cambridge Huntingdon Road Cambridge CB3 0JG UK; ^2^ Behavioural Ecology Research Group, School of Life Sciences Anglia Ruskin University East Road Cambridge CB1 1PT UK; ^3^ Computing Informatics and Applications Research Group, School of Computing and Information Sciences Anglia Ruskin University East Road Cambridge CB1 1PT UK; ^4^ Pattern Recognition Lab, Department of Computer Science Friedrich‐Alexander‐Universität Erlangen‐Nürnberg Erlangen 91058 Germany; ^5^ Université de Toulon, Aix Marseille Univ, CNRS, LIS, ILCB, CS 60584 Toulon 83041 CEDEX 9 France; ^6^ K. Lisa Yang Center for Conservation Bioacoustics, Cornell Lab of Ornithology Cornell University 159 Sapsucker Woods Road Ithaca New York 14850 USA; ^7^ Biology Department Carthage College 2001 Alford Park Dr, 68 David A Straz Jr Kenosha Wisconsin 53140 USA; ^8^ African Institute for Mathematical Sciences 7 Melrose Road, Muizenberg Cape Town 7441 South Africa; ^9^ Stellenbosch University Jan Celliers Road Stellenbosch 7600 South Africa; ^10^ African Institute for Mathematical Sciences ‐ Research and Innovation Centre District Gasabo, Secteur Kacyiru, Cellule Kamatamu, Rue KG590 ST No 1 Kigali Rwanda; ^11^ Centre of Ecology and Conservation, College of Life and Environmental Sciences University of Exeter, Cornwall Campus Exeter TR10 9FE UK; ^12^ Wildlife Conservation Research Unit Recanati‐Kaplan Centre Tubney House, Abingdon Road Tubney Abingdon OX13 5QL UK; ^13^ Department of Computer Science University of Oxford Parks Road Oxford OX1 3QD UK; ^14^ University of Oviedo Mieres Principality of Asturias 33600 Spain; ^15^ School of Natural Sciences, University of Lincoln Joseph Banks Laboratories Beevor Street Lincoln Lincolnshire LN5 7TS UK; ^16^ Institute of Environment Education and Research Pune Bharati Vidyapeeth Educational Campus Satara Road Pune Maharashtra 411 043 India; ^17^ Department of Zoology, Faculty of Science Charles University Prague 128 44 Czech Republic; ^18^ Institute of Zoology Zoological Society of London Outer Circle London NW1 4RY UK; ^19^ Tilburg University Tilburg The Netherlands; ^20^ Naturalis Biodiversity Center Darwinweg 2 Leiden 2333 CR The Netherlands; ^21^ Department of Archaeology University of Cambridge Downing Street Cambridge CB2 3DZ UK; ^22^ Department of Behavioral and Cognitive Biology University of Vienna, University Biology Building (UBB) Djerassiplatiz 1 Vienna 1030 Austria

**Keywords:** animal communication, artificial intelligence, automatic detection, bioacoustics, classification, deep learning, machine learning, neural networks, passive acoustic monitoring

## Abstract

Recent years have seen a dramatic rise in the use of passive acoustic monitoring (PAM) for biological and ecological applications, and a corresponding increase in the volume of data generated. However, data sets are often becoming so sizable that analysing them manually is increasingly burdensome and unrealistic. Fortunately, we have also seen a corresponding rise in computing power and the capability of machine learning algorithms, which offer the possibility of performing some of the analysis required for PAM automatically. Nonetheless, the field of automatic detection of acoustic events is still in its infancy in biology and ecology. In this review, we examine the trends in bioacoustic PAM applications, and their implications for the burgeoning amount of data that needs to be analysed. We explore the different methods of machine learning and other tools for scanning, analysing, and extracting acoustic events automatically from large volumes of recordings. We then provide a step‐by‐step practical guide for using automatic detection in bioacoustics. One of the biggest challenges for the greater use of automatic detection in bioacoustics is that there is often a gulf in expertise between the biological sciences and the field of machine learning and computer science. Therefore, this review first presents an overview of the requirements for automatic detection in bioacoustics, intended to familiarise those from a computer science background with the needs of the bioacoustics community, followed by an introduction to the key elements of machine learning and artificial intelligence that a biologist needs to understand to incorporate automatic detection into their research. We then provide a practical guide to building an automatic detection pipeline for bioacoustic data, and conclude with a discussion of possible future directions in this field.

## I. INTRODUCTION

### (1) Acoustic monitoring

The acoustic monitoring of captive and wild animals provides valuable data for many purposes, including scientific research, conservation efforts, management decisions, and the welfare of individual animals. Acoustic data can be collected using handheld microphones, on‐animal devices, or autonomous recording units (ARUs) placed in the field. Such data can be collected over periods of time ranging from short, opportunistic recordings to long‐term deployments lasting months or years. Handheld microphones and ARUs are non‐invasive methods that do not require the capture of individual animals, and so reduce disturbance and welfare impacts (Browning *et al*., 2017; Soulsbury *et al*., 2020; Ross *et al*., 2023). Acoustic data can help with the monitoring of elusive, cryptic, or nocturnal species that are difficult to observe directly (Zwerts *et al*., 2021), such as bats (Frick, 2013), wolves *Canis lupus* (Harrington & Mech, 1982; Kershenbaum, Owens & Waller, 2019), or marine mammals (Fleishman *et al*., 2023). Additionally, where animals use long‐distance vocalisations, ARUs are beneficial in recording species over large spatial scales, for example crested argus pheasants *Rheinardia* spp. (Vu *et al*., 2023), gibbons (Vu & Tran, 2019; Dufourq *et al*., 2021), howler monkeys *Alouatta* spp. (Pérez‐Granados & Traba, 2021), wolves (Kershenbaum *et al*., 2019; Smith *et al*., 2021) or cetaceans (Zimmer, 2011). Such methods can offer detection ranges on the order of several kilometres for some species, compared with tens of metres for camera traps. However, as a passive technique, the obvious disadvantage of acoustic monitoring is that the animal needs to be producing sound to be detected.

Whilst the collection of acoustic data offers many benefits and opportunities, it brings with it certain challenges. First, the deployment and servicing of ARUs (e.g. replacing batteries and memory storage cards) can be costly in terms of time and labour (Metcalf *et al*., 2023). Second, although the tools for acoustic monitoring are now more widely available, cheaper in cost, and include larger storage capacities and longer battery life (Hill *et al*., 2019), this has led to a very large increase in the quantity of data being stored, transferred, and analysed. Third, a major challenge is distinguishing the sound(s) of interest from background sounds which takes an enormous amount of researcher time, effort, and expertise, to recognise the calls of species accurately and annotate the recordings reliably. All of this creates long delays between data collection and the final results of a study, yet the need for real‐time results can be pressing, especially in the field of conservation biology. Automatic detection can solve many of these issues, as a tool to extract sounds of interest automatically, reducing or even eliminating the need for manual analysis of the data.

#### (a) What is automatic detection?

Throughout this paper, we will use the term “acoustic signal” to describe any sound or acoustic event produced by an animal without regard to the purpose or intentionality of the signal. This category includes vocalisations (calls, song) as well as non‐vocal sounds such as stridulation. Automatic detection is the process of identifying the presence of acoustic signals from sound recordings automatically, without human effort. Once detected and further processed, numerous properties of the acoustic signal can be determined (with or without additional human effort). For example, the acoustic signal could be classified as being produced by a particular species, its location determined, and the identity of the animal inferred. The temporal and spectral properties (e.g. call rate, fundamental frequency, harmonics, modulation, etc.) of the acoustic signal can be measured and used for additional processing or for inferring additional information about the sound production. Some approaches implicitly combine the processes of automatic detection with other tasks, for example classification of the detected species, but, fundamentally, the first step within an automated bioacoustic processing pipeline is detection.

#### (b) Scope of the review

This review arose from an investigative workshop held in July 2023 at Girton College, University of Cambridge, attended by 22 scientists from both the biological and computer sciences. In this review we set out to highlight and describe the emerging field of automatic detection of acoustic signals as a highly interdisciplinary effort that requires expertise from both biological and computer science to move forward. We present a review and tutorial that addresses both the needs of the community of biologists using acoustic monitoring to answer ecological, evolutionary, and conservation research questions, and the needs of computer scientists developing new algorithms and implementations. As the overlap between these two needs and the overlap between domain knowledge of these two groups is often small, this review attempts to bridge that gap by addressing both groups simultaneously, emphasising the missing knowledge of both. A reader from either field will find this review to be a useful integration of both domains, providing new information to both without being inaccessible to either. Whilst we acknowledge that there are ethical concerns surrounding the recording of human activities (Sharma *et al*., 2020; Sandbrook *et al*., 2021), and that automatic detection and removal or masking of human speech could alleviate some of these concerns (e.g. Cretois, Rosten & Sethi, 2022), we focus this review on the automatic detection of animal sounds and encourage more in‐depth discussion of human detection elsewhere.

By way of introduction, the review first presents perspectives on automatic detection for bioacoustics from the point of view of a biological researcher, aiming to instruct the computer scientist in the needs of the end‐user. Then, we present the perspective of the computer scientist, aiming to instruct the biologist in the technologies available and their limitations. There then follows a step‐by‐step guide to the practical implementation of automatic detection, and finally a discussion of the potential future directions of this field.

Several existing reviews of automated detection for passive acoustic monitoring (PAM) have been developed in recent years. Certain existing reviews have a narrow focus on either a specific taxon, such as birds (Pérez‐Granados & Traba, 2021; Xie *et al*., 2023), insects (Kohlberg, Myers & Figueroa, 2024), or cetaceans (Usman, Ogundile & Versfeld, 2020; Kowarski & Moors‐Murphy, 2021), or emphasise specific applications, such as welfare monitoring of livestock (Mcloughlin, Stewart & McElligott, 2019). Others do not comprehensively explore machine learning (ML) (Gibb *et al*., 2019; Sugai *et al*., 2019; Mutanu *et al*., 2022) or are very focused on a particular field of ML, for example classical methods (Bittle & Duncan, 2013) or deep learning (DL) methods (Stowell, 2022). Our review distinguishes itself from the existing literature by providing a comprehensive and interdisciplinary roadmap tailored for both biologists and computer scientists. This is crucial as existing reviews often provide limited guidance to research students new to the concepts in either PAM or automatic detection, whereas ours aims to fill this gap, making it an ideal starting point for newcomers to the field. Additionally, while Sharma, Sato & Gautam (2022) present a related but brief overview including 20 studies, our review extends beyond this by encompassing the broader field of automatic detection for PAM. By integrating these perspectives, our review updates the academic community on the broader advancements of automated detection and serves as a practical guide for emerging scientists in this rapidly evolving field.

## II. BACKGROUND TO AUTOMATIC DETECTION IN BIOACOUSTICS

### (1) What is automatic detection and why do we need it?

**Fig. 1:**
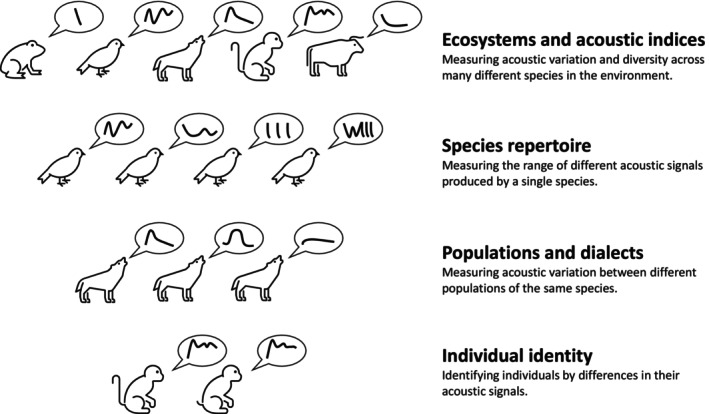
Hierarchy of acoustic signal specificity.

To address the challenge of converting terabytes of acoustic recordings into useful information, scientists have sought to develop techniques to automate the detection of acoustic signals of interest. The traditional method of identifying the signals of interest from longer acoustic recordings was to create a spectrogram and manually draw bounding boxes around the signals of interest, incurring a significant cost in terms of time and expertise. Fundamentally, the challenge is to replace the human annotator with computational methods without a consequent loss in accuracy (Miller *et al*., 2023). At its simplest, the aim of automatic detection is to indicate segments or windows of audio that are likely to contain a target sound of interest, substantially reducing the burden, even if the automated annotations need then be checked by a human. The annotation label can simply be a binary label of presence/absence of a sound, but this can also be further refined to classify by taxon, call‐type, number of individuals, etc., in increasing levels of precision and consequent difficulty for both annotator and algorithm (Fig. 1). For some species, it can be possible to identify an individual through its unique vocal characteristics (Petso, Jamisola & Mpoeleng, 2021). In addition to the class label, it is possible to design PAM systems that also allow the position or bearing of the sound to be estimated (Kershenbaum *et al*., 2019; Smith *et al*., 2021). Such information can then be used in numerous downstream tasks such as occupancy monitoring, spatial habitat use, and behavioural analysis, and automatic detection offers researchers the opportunity to scale to larger spatiotemporal data sets.

### (2) The current state of the art in automatic detection

The use of automatic detection to accelerate acoustic monitoring has a relatively long history (Anderson, Dave & Margoliash, 1996; Acevedo *et al*., 2009; Aide *et al*., 2013; Dufourq *et al*., 2021; Oswald *et al*., 2022). As an early approach towards automation, simple techniques based on the energy within a particular frequency range, characteristic to the target sound, were used to detect signals of interest (Ward *et al*., 2000). More sophisticated approaches consider the relative energy across multiple frequency bands obtained through spectral or wavelet decomposition, which provides higher robustness to variable background noise (Gillespie & Chappell, 2002). However, these approaches only work if the signal‐to‐noise ratio (SNR) of the target sound is sufficiently high, and if other sounds are not present in the same frequency range which act to mask it.

Other approaches are based on the use of a template or kernel which acts as a prototypical example vocalisation (Mellinger & Clark, 1997). Through the use of either one‐dimensional (1‐D; temporal) or two‐dimensional (2‐D; spectrogram) cross‐correlation, a score can be derived which captures how closely the recorded sound matches the template sound. By careful selection of a threshold (which may need to be dynamic), detections can be produced. These template‐based approaches are efficient and easy to understand, but do not generalise well to variations in call structure and struggle to discriminate between calls from different species with similar structure.

Subsequent techniques have used statistical modelling such as hidden Markov models to detect calls that are modulated in frequency and/or time (Duan *et al*., 2013; Oswald *et al*., 2022), by identifying properties of the target sound beyond simply frequency range. Such models can provide more robust and sensitive detections. More recently, there has been a strong push towards the use of data‐driven ML, exemplified by DL, using techniques such as convolutional neural networks (CNNs) (LeCun, Bengio & Hinton, 2015), recurrent neural networks (Yu *et al*., 2019) and more recently transformers (Lin *et al*., 2022). CNNs have been shown to obtain impressive detection accuracies, for example BirdNET (Kahl *et al*., 2021), and the BTO Acoustic Pipeline (British Trust for Ornithology, 2023). [Correction added on 27 November 2024, after first online publication: “Transformers” has been corrected to “CNNs.”] Note, however, that advances in DL are built on foundations such as correlation/convolution, simply with large numbers of stacked, learnable kernels and non‐linearity between layers.

There is, however, a highly fragmented landscape in the field of automatic detection – in particular between the fields of computer science/ML, and bioacoustics/acoustic ecology – and it can be very challenging for practitioners to know where to get started. Should one build their own classifier from scratch, fine‐tune an existing model, or simply use an off‐the‐shelf pretrained model (Dufourq *et al*., 2022; Stowell, 2022)? Good quality detectors already exist in a relatively user‐friendly format for: birds (e.g. BirdNet; Kahl *et al*., 2021), bats [e.g. BTO Acoustic Pipeline (British Trust for Ornithology, 2023); Kaleidoscope (Wildlife Acoustics, Inc, 2024)], cetaceans (e.g. PAMGuard; Gillespie *et al*., 2009) and rodents [e.g. DeepSqueak (Coffey, Marx & Neumaier, 2019); MUPET (Van Segbroeck *et al*., 2017)]. However, these detectors tend to be known only by those using them in the field and are not straightforward to generalise to other taxa without retraining or altering the model architecture or assumptions. There is also an imbalance with some taxa being better represented than others in terms of the availability of detectors. The process of building or fine‐tuning a new DL model for a practitioner's particular habitat and species of interest is non‐trivial and involves several tasks such as installing the correct software scripts, designing data‐loaders, and training models on specialised computing hardware such as Graphical Processing Unit (GPU) clusters. This serves as a major barrier to widespread adoption of these new techniques, unless a tame computer scientist can be persuaded to assist in the process. By contrast, the more mature field of automatic detection in image‐based detection (“camera trapping”) [e.g. Camelot (Hendry & Mann, 2018) and Agouti (Casaer *et al*., 2019)], can serve as an exemplar for deriving best practices, as existing tools are easy to use for non‐programmers, and easily generalised to different taxa.

### (3) What do we hope for from automatic detection?

Despite the challenges associated with the automatic detection of acoustic signals, rapid advances in ML are starting to bring this concept into reality. Although the context under which acoustic data are recorded and their end use will vary widely, the common requirement is for algorithms that take acoustic data as an input, and then detect and return extracted sounds as the output. Some users may only require outputs of particular target sounds, such as a selected species, whereas others may require all sounds of potential interest to be detected. Ideally, the ultimate end goal of automatic detection for biologists would be a universal, off‐the‐shelf algorithm capable of detecting and classifying all animal vocalisations such that anybody, including those without any training in computational methods, could process their acoustic data more efficiently and tailor it flexibly to their particular use‐case (Romero‐Mujalli *et al*., 2021). Where an off‐the‐shelf detector for a sound of interest is not readily available, algorithms that are easy to train with a relatively small amount of data and minimal annotation effort should be the aim.

## III. PERSPECTIVES FROM BIOLOGICAL SCIENCES

In this section, we provide, largely for the benefit of the reader from a computer science or other non‐biological background, an overview of the possible roles for bioacoustics in addressing several important evolutionary, ecological, and conservation questions, highlighting the potential benefit that automatic detection can provide.

### (1) Overview of uses of automatic detection in the biological sciences

Detecting acoustically active animals through their acoustic signals can provide a wealth of information that is important to conservation biology, ecology, evolutionary biology, animal behaviour, and welfare (Mcloughlin *et al*., 2019; Odom *et al*., 2021; Erbe & Thomas, 2022). Often, these areas of study can overlap: animals can produce sounds to influence the behaviour of others in a wide range of contexts, for example to attract a mate or warn off an intruder, or as a by‐product of other behaviours, such as the sound of wings flapping or footsteps.

Historically, conservation efforts and biodiversity surveys have been skewed towards species that are easy to trap or track across the landscape, often depending on direct observation or finding physical traces, such as scat or hair (Boakes *et al*., 2010). However, the field of bioacoustics allows us to survey remote or otherwise inaccessible areas without the need for the researcher to be present, for example deep sea environments, Arctic and Antarctic regions, and rainforests (Staaterman *et al*., 2017), with research often focusing on the loud and persistent calls of target species to detect their presence. Like image‐based detection, bioacoustics generates large data sets which challenge analysis, but, unlike camera traps, the same sound can be recorded in multiple places, multiplying the data to be assessed and analysed.

Below, we provide a broad review of the uses of acoustic data in the biological and ecological sciences, from measures of biodiversity at geographic scales to tracking the movements and behaviours of individual animals, and highlight how automatic detection can increase the efficiency and efficacy of monitoring.

#### (a) Ecosystems and acoustic indices

Any multi‐species soundscape will consist of a wide range of frequencies being used by different species in the same environment (Krause, 1993). To maximise the chance that their signal will be audible to others, animals usually avoid acoustic signal interference by vocalising in different frequency ranges or at different times, as described by the acoustic adaptation hypothesis (Hansen, 1979; Rothstein & Fleischer, 1987). This ecological phenomenon makes it possible to detect particular clades or species. It also means that estimates of biodiversity can be made based on the number of different acoustic signals being produced at different times/frequencies.

Acoustic indices are summary metrics that provide a quantitative measure of acoustic complexity by analysing variation in the frequency and timing of acoustic signals, rather than identifying individual sounds. Such indices offer metrics for wildlife monitoring and assessment, characterising biological communities through sound (Sueur *et al*., 2014; Buxton *et al*., 2018; Alcocer *et al*., 2022). While acoustic indices are informative about the acoustic complexity or general biodiversity of a landscape, they are less useful for deriving specific information about species or the individuals vocalising.

Acoustic indices typically do not use automatic detection and classification of acoustic signals, as, by their nature, they characterise the soundscape as a whole. However, automatic detection of sound classes, for example distinguishing acoustic signals of anthropogenic origin from those of biological origin, can improve the ability of acoustic indices to provide useful indications of biological activity (Narasimhan, Fern & Raich, 2017; Fairbrass *et al*., 2019; Clark *et al*., 2023). Thus, effective automatic classification of acoustic signals may become an important element of improving acoustic indices in future research.

#### (b) Species occupancy and density

Occupancy modelling is the statistical analysis of the patterns and dynamics of a species in a given space over time (MacKenzie *et al*., 2003), which can be informed by acoustic signals (Wood & Peery, 2022; Cole *et al*., 2022). Bioacoustic occupancy monitoring can provide critical information on the presence and absence of species and the dynamics of the ecosystem, particularly for cryptic or elusive species.

Population density estimates model a species' abundance within a defined space. Density estimates are an extremely important tool for assessing spatiotemporal population changes that can be the result of declining prey numbers, land‐use change, human–wildlife conflict (Wolf & Ripple, 2016; Ogutu *et al*., 2016), or other factors, and bioacoustics data can provide an important tool for estimating the densities of animal populations (Marques *et al*., 2009).

#### (c) Spatial analyses

Population surveys and behavioural research often need to be able to determine the location and/or movement patterns of animals. Bioacoustic surveys have been used in more recent years to supplement or replace previous tracking methods (Frommolt & Tauchert, 2014). For example, the tracking of migratory species across their extensive ranges, where radio/satellite telemetry is only useful if the individuals tagged with a transmitter survive what may be a high‐mortality journey, can benefit from the application of bioacoustic techniques. While telemetry is an effective method for learning about a species' movement, it can also be highly invasive, can affect the behaviour of individuals being trapped, and is not always suitable for all species/age groups, for example species that are too small to carry the weight of a transmitter, or species in remote areas (Sharpe *et al*., 2009).

Depending on the intended research goals, it may be sufficient simply to detect the presence/absence of an animal within a recorder's range (macro‐localisation), or one may need to infer the exact position of an individual (micro‐localisation). There are benefits and limitations to each: macro‐localisation can inform on occupancy, habitat suitability, territory use, and migratory patterns. On the other hand, with a significant increase in the complexity of use, advanced tools also allow a more targeted approach such as multilateration, where the exact individual's location is calculated based on the time difference of arrival (TDOA) of an acoustic signal to multiple recording devices (Mennill *et al*., 2012). Such micro‐localisation avoids double counting (if that is required) for density estimates, can inform on animal movement speed and direction, as well as providing fine‐grained territory boundaries, but requires additional downstream processing to carry out the localisation analysis.

Estimation of a focal animal's home range and territory can provide wildlife managers with a boundary for their activity (Powell, 2000), permitting the study of intraspecific dynamics and spatial distribution of individuals across a landscape (Burgos & Zuberogoitia, 2020), which can be important for conservation action. The feasibility of using PAM to monitor animal ranging patterns will depend on the natural history of the animals, available resources, and ethical concerns For example, PAM has been used to estimate home range size in bats (Coleman *et al*., 2014) and to monitor spatial activity in chimpanzee *Pan troglodytes* communities (Kalan *et al*., 2016). The use of global positioning system (GPS) collars or satellite tags may provide more precise estimates of ranging behaviour, however, for many species (e.g. those where for conservation reasons, capture and sedation is undesirable) these approaches are not feasible, warranting a non‐invasive approach such as PAM. Real‐time automatic detection combined with localisation reduces the research effort required for follow‐up visual observation and can obviate the need for visual observation entirely.

#### (d) Species characteristics

**Fig. 2:**
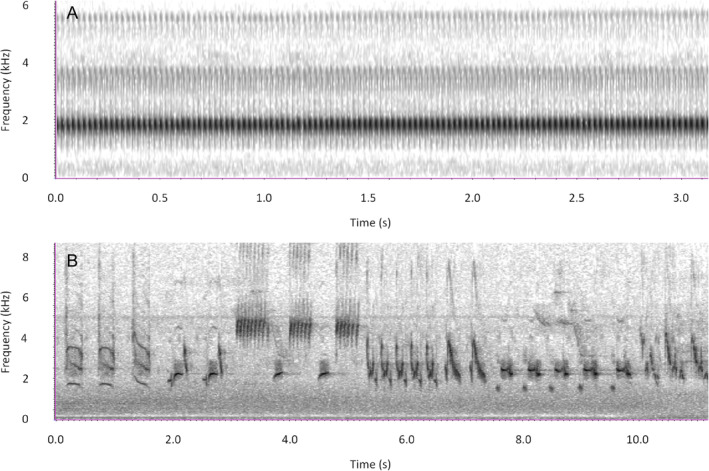
(A) The call of the American toad (*Anaxyrus americanus*), which only produces a single call, repeated for long periods. (B) The varied mimicry of the northern mockingbird (*Mimus polyglottus*), composed of varied songs of other species, which would be difficult to detect in a general way. The spectrograms show time on the *x*‐axis and frequency on the *y*‐axis.

Automatic detection of acoustic signals is complicated by the fact that there are relatively few species that, like the American toad (*Anaryxus americanus*), produce a single call (Bee, 2012), while many species produce multiple call types [e.g. the northern mockingbird (*Mimus polyglottos*) produces hundreds of different song types (Derrickson, 1988)] (Fig. 2). Thus, while it is relatively easy to link a croak to the presence of a toad, it can be more challenging to design a tool that will detect all the potential acoustic signals of the mockingbird. This is further complicated if the species' different call types need to be classified beyond simple detection (e.g. as contact calls *versus* alarm calls).

Collectively, all the distinct call types a species produces can be defined as the vocal repertoire. The size of the repertoire may be thought of as a simple proxy for vocal complexity (Bouchet, Blois‐Heulin & Lemasson, 2013; Manser *et al*., 2014), and the structure of the repertoire (e.g. how often call types are used and interpretations of the potential uses) are important for describing a species' behavioural ecology. Therefore, both general acoustic signal detection (“the target species made a sound in some way”) and specific call‐type detection (“the leopard‐specific alarm call has been produced”) are useful to different studies and these analyses can be nested. Comparisons of vocal complexity between species, species groups, and taxa (Kershenbaum *et al*., 2021; Leighton & Birmingham, 2021) may enable research into broad evolutionary or ecological questions, such as cognitive abilities, adaptive advantages of cognitive skills, or the evolution of language (McComb & Semple, 2005; Dunn & Smaers, 2018).

The more varied and less stereotyped calls are, the larger the challenge to automatic detection. However, the implications of variability within a single call type on the performance of automatic detection and classification have not been adequately investigated.

#### (e) Populations and social groups

The same species can show variation in their vocalisations among social groups and or across geographic regions. Research into these differences can offer unique insight into phylogenetic patterns, speciation (Meyer *et al*., 2012; Riesch *et al*., 2012; Heaphy & Cain, 2021), historic geographical patterns (Laiolo *et al*., 2001; Kershenbaum *et al*., 2012; Hebets *et al*., 2021), or differences between social groups (Ford, 1991; Velásquez *et al*., 2013; Garland, Castellote & Berchok, 2015; Kershenbaum *et al*., 2016*b*). Automatic detection can scan through long‐term recordings to unveil temporal and cultural variations of vocal behaviours, for example in whales (McDonald, Hildebrand & Mesnick, 2009; Garland *et al*., 2011; Best *et al*., 2022).

#### (f) Individual characteristics

For some research questions, it may be important to identify individual animals and/or characterise the traits or states of individuals of a target species, such as age, sex, body size, emotional valence/arousal, and physiology. Acoustic signals can potentially encode all of this information. Examples of the benefits of individual identification include gaining insights into the evolution and ecology of a species, such as life‐history stages and social structure (Clutton‐Brock & Sheldon, 2010); facilitating conservation efforts, for example tracking movement of critically endangered species in the landscape (Mcloughlin *et al*., 2019); and improving management in captivity, for example measuring vocal activity as an indicator of welfare in zoo‐housed animals (Castellote & Fossa, 2006; Clark & Dunn, 2022). A diverse range of species' calls have been found to encode individual identity from birds (Fox, Roberts & Bennamoun, 2008; Martin *et al*., 2022) to cattle (Green *et al*., 2019), cetaceans (Kershenbaum, Sayigh & Janik, 2013; Bøttcher *et al*., 2018), and frogs (Qian *et al*., 2023). Individual identification provides an open scope for spatiotemporal monitoring of species without tagging (Aide *et al*., 2013), while also offering the opportunity for population estimation using mark–capture recapture methods, which rely on individual identification (Marques *et al*., 2013; Buxton *et al*., 2018).

Acoustic signals can be used in a wide range of species to assess the intensity (high to low) and valence (positive to negative) of emotional arousal of animals, which in turn can be used as an estimate of welfare in animals in captivity (Volodina & Volodin, 1999; Clark & Dunn, 2022) and farms (Manteuffel, Puppe & Schön, 2004). Inferring emotional arousal from acoustic signals also allows for the assessment of “positive welfare” in animals (Laurijs *et al*., 2021), and it is possible to monitor farm animals for the onset of disease [e.g. pigs (*Sus domesticus*) (Exadaktylos *et al*., 2008; Mcloughlin *et al*., 2019) and chickens (*Gallus domesticus*) (Mao *et al*., 2022)].

### (2) Key challenges

As outlined above, many studies in ecology and evolution require relatively precise identification of the type of acoustic signal, for example different call types, the source of the sound, individual identification, or the localisation of the source of the sound in space. Despite the huge potential for automatic detection to answer these challenges, the field is still facing significant barriers during implementation in biological studies, ranging from limitation in infrastructure, lack of training, inaccessibility of methods, and practical limitations in the field. For example, field recordings are often not of optimal recording quality and have a low SNR. Even under ideal conditions, acoustic signals themselves may be highly varied and irregular, with low stereotypy and a high degree of variability between individuals and groups, or geographical dialects (Nelson, 2000), all of which can present a challenge for automatic detection. The broad uptake and implementation of automatic detection requires that models are robust to such variation.

The training of models requires data to be robustly identified and correctly attributed to the study species or individual, often derived from visual observation of the callers. Collecting these data can be challenging as, for instance, individuals may remain visually cryptic, or call only at certain times. Thus, ground‐truthing data requires high quality, reliably identified call data sets which can be difficult to obtain, but are essential. Furthermore, generalising data from captive animals or in unique circumstances might give rise to misleading results (e.g. owing to differences in call structure or repertoire). Thus, robust ground‐truthing of large data sets is rare, but essential, and should be a focus for future research.

## IV. TECHNICAL PERSPECTIVES

### (1) Perspectives from computer science

#### (a) The role of computation in automatic detection

Advanced computational methods can provide solutions to a wide range of bioacoustic problems. For example, acoustic signals of interest can be merely detected (i.e. the start and end times identified), or additional information can be extracted, such as classification of signal type, or location of the sound source. If different types of acoustic signal are present, they can be grouped into multiple classes, which might represent different species, or different call types within a single species. Even when a single type of acoustic signal is present, the task of counting the number of such events or sub‐elements of the events is often non‐trivial (e.g. the different notes in a birdsong, or the individual barks of a dog). Therefore, the role of automatic detection and automatic processing of bioacoustic data is a broad field, with many possible applications.

Computational methods can help with any task that can be clearly defined. One way to define the task is through explicit rules (an engineering approach), for example, to specify that a target acoustic signal occurs solely and uniquely in a certain range of frequencies. Alternatively, a set of examples can be provided to the algorithm (a ML approach), and the algorithm is trained to generalise those examples to detect successfully when presented with novel examples. In the case of automatic detection, some tasks are simple enough that a good method can be designed directly using the engineering approach: this typically happens with situations of highly stereotyped sounds, where template‐matching often works well (Barker, Herrera & West, 2014), or low‐noise environments with few interfering sounds, where energy detection may work well (Hood, Flogeras & Theriault, 2016).

When the target sounds, or the background, are more complex – such as with recordings of elaborate bird song or soundscapes with high levels of anthropogenic noise – then ML is of benefit. As noisy problems can rarely be defined in a clear‐cut “engineering” way, ML attempts to reach a solution by generalising from a set of examples instead. Although ML has been investigated for many years (Towsey *et al*., 2012), it is the era of DL that now makes many bioacoustic detection tasks achievable (Stowell, 2022). It is still important to define the task to be solved clearly – by curating good data sets for training and evaluating systems, and by specifying the input and output data formats. Input data format, in bioacoustic applications, is generally some representation of the sounds recorded, whereas the output format is defined by the nature of the “answer” that the system is trained to supply, for example species presence, individual, call type, etc., or properties of the call itself.

Data curation aside, the power of ML comes from having techniques that can “train” (optimise) the system to achieve a particular goal, and so the output data format matters because it is closely tied to this procedure of optimisation. If the output format is a yes/no answer about species presence, this is the same format as a binary classification task in ML and can be addressed directly by training a classifier (Stowell, 2022), which takes sound as input, and outputs a corresponding indicator: present/absent. Very often, however, the output format wanted is more complex; for instance, given a long audio recording as input, we may want to output a list of (predicted) events, giving each event's start and end time, and optionally its frequency range as well. Note that this is quite similar to “object detection” in image recognition, and indeed, most bioacoustic research uses spectrograms as a visual representation of a sound, rather than working with the sound directly. In this case, we may typically be looking for a list of “bounding boxes” along the time axis or in time‐frequency, leading some directly to adapt image object detection algorithms to spectrograms (Kershenbaum & Roch, 2013; Venkatesh, Moffat & Miranda, 2022; Wu *et al*., 2022).

When a ML model has been trained, better results may be obtained if the model is applied in the same conditions as the training data, that is “in‐domain” as opposed to “out‐of‐domain” data (Best *et al*., 2020). For example, conditions might be “in‐domain” if they have the same background conditions, microphone type, and sampling protocol as in the training data, for example the same cetacean species in two different oceans.

#### (b) State of the art in automatic detection methods

No algorithm will generalise perfectly to all situations: the choice of training data represents the choice of intended domain. Classic ML advice would be to avoid “out‐of‐domain” situations, but many taxa do not benefit from such a large amount of prior work as that which has been carried out, for example, on birds. Could we nevertheless make use of off‐the‐shelf models from similar tasks, or must we start building a large new data set?

Happily, a recent widespread trend involves “transfer learning”, using one or more pretrained models that have been trained on tasks that are different from (but usually related to) the original domain: for example, we could consider models trained on human speech recognition. The models are then re‐used for the current application (i.e. acoustic signals of other species), and it is often found that the original learning makes training the model on the current data more effective (Zhuang *et al*., 2021).

A common approach to transfer learning, known as fine‐tuning, consists of modifying only a small subset of parameters and adapting the inputs and/or outputs. The modification requires training the model on a new set of examples, made up of audio recordings and corresponding annotations. This procedure is computationally much lighter than performing the process from scratch. It also requires fewer labels since it exploits many of the regularities in the initial data set. As a rule of thumb, one may try to choose a base model that has been trained on similar target sounds or background noise, for example BirdNet, an algorithm trained for birds that has been used for various bioacoustic tasks (Kahl *et al*., 2021). Yet we have observed successful attempts in adapting models from significantly different acoustic data, even from different frequency ranges, especially applying models trained on human data to broader ecological systems (Çoban *et al*., 2020; Sethi *et al*., 2020; Leroux *et al*., 2021; Sarkar & Magimai Doss, 2023).

When using transfer learning (also known as “pretrained” models), special care must be taken. The model must be applied to acoustic data that closely resemble the data on which it has been trained. The user must reflect on details such as matching sampling rates, normalisations, SNR‐levels, and duration of the input audio segments. Usually, the producers of such models will have trained models on diverse data to ensure generalisation. However, optimal performance is achieved when staying within the domain of operation for which the model was designed.

The algorithm trained on a different system can be considered to perform a role similar to the role of the spectrographic representation in aiding human interpretation of sounds. In the same way that a spectrogram or filterbank takes a sound waveform and presents it in a different format (and one where the important features are easy to detect by eye), so a model trained on a different species, for example, cannot detect the target species well, but may nonetheless produce an output (known as extracted acoustic features) that can be used as the input to train another model, which will then be more successful in finding the focal species. In the ML literature the resulting features are often referred to as embeddings or latent representations. Unlike traditional acoustic features like a spectrogram, these embeddings are often difficult to interpret on their own. They are the result of a large composition of complex functions whose parameters have been optimised to solve a particular task such as classifying an acoustic scene or discriminating from a given set of videos the one that matches a particular sound. Fine‐tuning alone may not be sufficient to obtain the desirable level of accuracy. We may then further adapt the model to our specific needs by retraining all of its parameters on the acoustic data of interest. One must take into consideration that these models have been designed with a large number of parameters – 317 million parameters for the large version of HuBERT for instance (Hsu *et al*., 2021) – to be optimised on thousands of hours of audio. When trained on a small number of examples this may quickly lead to overfitting, where the model will work as expected on the data presented during training but will fail to produce satisfactory predictions for unseen audio examples.

Even when many hours of field recordings are available, it is not clear if the acoustic data will be sufficiently diverse to produce acoustic features that will be performant for downstream tasks such as the detection of vocalisations. In other words, if a bioacoustic data set does not contain any useful (or additional) information which could be reemployed in the downstream detection tasks, then retraining the pre‐trained model might not improve performance. Furthermore, re‐training these models on large amounts of data is usually a tedious task which calls for the expertise of trained computer scientists and access to costly computational resources such as GPU clusters.

The approach of adapting transfer learning models to automatic bioacoustic detection can still be carried out by pretraining models on bioacoustic data directly, instead of human speech or generic audio. It has been shown to yield interesting results in the downstream detection performances for a variety of species (Hagiwara, 2022), but much work still needs to be done in this area. The success of this method relies on the availability of large data sets which could allow for the pretraining of a single, large‐scale, multispecies foundation model. As is the case in the speech processing and image recognition domains, making such a model available to the bioacoustic community could then allow for efficient user‐friendly classifiers to be trained for new tasks (“fine‐tuned” i.e. derived from the original trained system) within a unified pipeline.

An alternative to the transfer learning approaches is to use smaller models, with fewer parameters that may be trained entirely on the target audio data. For example, an algorithm called TweetyNet is designed for detecting/segmenting bird vocalisations in a laboratory context, based on a CNN to be trained specifically for each target bird; the package includes an interface to simplify that training process (Cohen *et al*., 2022); DeepSqueak can do the same for rodent vocalisations (Coffey *et al*., 2019). Those algorithms directly train the CNN as a classifier/detector. Another approach used by many in the bioacoustics community is to train a so‐called “auto‐encoder” on the data set of interest to extract deep feature representations from unlabelled data. This unsupervised approach consists in optimising a neural network to compress the data from an audio snippet into a numerical vector; this compression is intended to create a semantic representation of the data, following the principle that a semantic representation should be a good solution to the problem of highly compressing data. This technique has been applied to call categorisation in a variety of species (Sainburg, Thielk & Gentner, 2020; Best *et al*., 2023).

Even using such methods, it is common that bioacoustic data sets are not large enough to train an ML detector well, or that some categories/contexts are underrepresented in the training data. It is thus common (and recommended) to use “data augmentation” to assist with this: “new” training examples can be created by small modifications of existing ones. This has been widely investigated and found to improve performance, to a similar extent as the use of pretrained networks (Lostanlen *et al*., 2018).

The bioacoustics community faces complex scenarios with sound events potentially overlapping both in time and frequency (e.g. dawn chorus of birdsong) or with highly non‐stationary background noise (e.g. urban scenes). These require more advanced and specific solutions that tackle the problem of working with mixtures of sounds. Data‐augmentation techniques serve this purpose by artificially constructing similar data for which annotations can be created by design (Jansson *et al*., 2017; Zhang *et al*., 2018; Wisdom *et al*., 2020). These approaches have been applied to improve performance on up to 10 simultaneously calling bird species in a simulation study (Parrilla & Stowell, 2022) and in real recordings with significantly fewer simultaneous calls (Denton, Wisdom & Hershey, 2021; Bermant, 2021).

#### (c) Assessing pre‐existing models

The fast pace at which the ML community publishes new pretrained models renders them outdated quickly. The availability of accessible learning resources for some models makes them a go‐to solution for many practitioners, despite having been superseded by other options. Model publishers should document their work in a way approachable by non‐experts if they aspire to have an important impact on the bioacoustic community. On the other hand, users of these models may consult the latest benchmarks and challenges that target diverse applications of audio ML representations. For instance, HEAR (Turian *et al*., 2022) benchmarked multiple state‐of‐the‐art methods on a varied set of tasks in speech, music and environmental sounds. More recently BEANS (Hagiwara *et al*., 2022) proposed a benchmark specific to bioacoustics where representations are tested on detection and classification tasks of several species.

### (2) Conclusions on the technical constraints on the current uses, limitations and expectations of automatic detection

Automatic detection has been used for density estimation (McDonald & Fox, 1999; Marques *et al*., 2013), occupancy (Dawson & Efford, 2009; Abrahams & Geary, 2020), species presence (Obrist *et al*., 2010), and phenology, for example the start of breeding, or daily onset of song (Willacy, Mahony & Newell, 2015; Oliver *et al*., 2018). This technology can be used in conjunction with other non‐invasive monitoring methods such as camera traps, scat surveys, hair collection, and human observation (Long *et al*., 2012), providing additional information and allowing monitoring of otherwise cryptic species that might elude detection. There should be ongoing conversations between biologists and computer scientists, bidirectional and iterative, improving the survey quality, accuracy, and algorithm usability over time. Biologists can provide the ground‐truthing and validation of the use of automatic detection, while computer scientists can develop the system and work with them to improve the automatic detection system iteratively.

While we have argued for the widespread use of automatic detection systems, there are limitations, and these should be considered at the start of a project. Some of these are self‐evident: signals that do not rise above background noise will be lost as undetectable. Also, signals can be difficult to separate if they overlap with either intraspecific, interspecific, or unrelated sounds, as in the dawn chorus when birds sing with many overlapping, very similar elements, making extraction/detection of a single unit difficult. Data set sizes (for both training and deployment) may be a limiting factor. We have referred to data augmentation and denoising to account synthetically for data limitations. These and other tools (e.g. data imputation) are often helpful, but the results are unlikely to be as reliable or unbiased as they would be with a large representative data set. They should not be relied upon as a silver bullet when recordings are rarely observed, noisy, or otherwise hard to analyse. Just as with human annotation, automatic detection will always be subject to some level of bias and inaccuracy; one advantage of automatic systems is that these factors can be numerically evaluated. Automatic detection model predictions are only ever as good as the input training data. Annotations that are not accurate or have not been conducted appropriately for the intended application may worsen the efficacy of the model. Furthermore, the usage of automatic detection systems should be made in awareness of error rates. Indeed, while tuning the confidence threshold enables balancing between precision and recall, it never completely removes false positives nor false negatives. Nonetheless, if error rates are correctly taken into account in the following analysis, yielded results will be reliable (e.g. density estimation is possible despite 50% of false positives if they are accounted for; Marques *et al*., 2009). There can be an accumulation of errors over time if the thresholds are chosen either to be too low or too high, discarding weak identifications wrongly, or placing too much confidence in others. Finally, all acoustic detection relies on the sound event occurring, and often animals may choose not to vocalise or create a sound and thus can be missed. What is not heard cannot be counted. However, despite these caveats, we believe that automatic detection and PAM offer the opportunity to collect and analyse data that cannot be processed by other means, providing an exciting and valuable new tool for the biological sciences.

## V. A PRACTICAL GUIDE TO AUTOMATIC DETECTION

We now present a practical guide for using automatic detection. There are many decisions that we must make when designing a study that uses automatic detection, and our goal is to help practitioners optimise these decisions. We realise that some of these decisions may be constrained by access to financial resources, lack of training in bioacoustics, limited technical skills in coding and ML, and/or lack of access to high‐speed internet for cloud storage and computing. These limitations may be particularly pronounced for researchers in the Global South. We acknowledge that there is still much to be done to make these tools and approaches accessible for all.

This guide is developed to help users implement an “off‐the‐shelf” automatic detection approach, or for developing or adapting their own approach. We strongly advocate that practitioners implement a pilot study to ensure the approach they plan to use is feasible before embarking on a large‐scale endeavour. Importantly, even with the most sophisticated automated approach, a substantial amount of human investment is needed to create training data sets, evaluate detector performance, and verify the detections.

### (1) Define research questions

The most important thing to consider when using automatic detection is the specific research question. For example, if you are interested in detecting the presence or absence of a rare acoustic signal (e.g. a gunshot or the presence of an endangered species) then you will want to use an approach that will ensure high recall (i.e. high probability of detection) and you may tolerate a relatively high number of false positives. Alternatively, if you are interested in subsequently classifying individuals from the detections, you may prefer to focus on retaining high SNR calls and will tolerate lower recall with higher precision. Your research question will influence every decision you make in the automatic detection workflow, including study design, data collection and the analytical approach. For guidance on defining research questions, we refer readers to Sugai *et al*. (2019).

### (2) Study design

Depending on the nature of the research question, researchers will need to determine their study design, including hardware needs, recording schedule and whether the processing of data will be carried out in real time or at a later date. For instance, for the detection of a single species, researchers may deploy ARUs over the landscape for a period of time and then download the data onto a hard drive to be processed offline. The recording schedule also needs to be determined according to the research goal. We refer the readers to more extensive discussions of this issue for further details (e.g. Browning *et al*., 2017; Metcalf *et al*., 2023). Real‐time processing is an emerging area, but due to the limitations of placing power‐efficient computation in the field, real‐time automatic detection typically is more bespoke and less accurate than offline processing.

### (3) Start with a pilot study (if possible)

Given the costs, both financial and in human labour, of implementing projects that use automatic detection, we strongly advocate that researchers start with a small‐scale setup to test out their planned approach. For a large‐scale PAM study, deploying a few recorders over a smaller spatial scale and a shorter time period may provide enough acoustic data to get started with automatic detection. If the signals are relatively rare (e.g. gunshots) perhaps finding online repositories or data sets of samples would be necessary. A well‐designed pilot study will help researchers make informed decisions about annotations, choosing an automated detector, and reporting and interpreting their results.

### (4) Data collection and archiving

Data storage and archiving remains challenging, since the large data volume of the raw audio in many projects often goes beyond the limits of free or easily available services. Furthermore, Metcalf *et al*. (2023) recommend backing up audio data in multiple copies, and also making use of cloud storage. Simply storing the audio is typically only part of the issue: you and your collaborators will also need to access it, for example to visualise or to apply an algorithm to the data set, which means that speed of upload and download (bandwidth) may be an equal or greater concern. Cost of storage and bandwidth are often significant questions. Arbimon (Ganchev, 2020) is one project that aims to store and share large volumes of wildlife audio on behalf of others.

Reducing data sizes can be achieved in many ways, including audio file compression and data subsampling. Lossless compression (such as FLAC) can reduce file size without losing information; lossy compression (such as MP3 or AAC) will discard at least some information from the signal, but might still support reliable analysis (Heath *et al*., 2021), depending on the research question. An alternative strategy very relevant in automatic detection, is to keep only the audio corresponding to the positive detections: for rarely occurring sounds this will greatly reduce the storage requirements, while keeping the detected audio clips available for inspection or re‐analysis. However, any missed (false‐negative) sound events will be irretrievably lost. This would also prohibit future interrogation of the raw data for other potential uses.

Good‐quality metadata including time, date, and location, is crucial for the success and reproducibility of any project. This can be stored in the audio files (as “RIFF tags”) or separately (Metcalf *et al*., 2023). Research and other publicly shared data should be “FAIR” – findable, accessible, interpretable, reusable (Wilkinson *et al*., 2016) – and publishing metadata in standardised formats is key to this. The Biodiversity Information Standards (TDWG) group maintains the metadata standards Darwin Core (Darwin Core Task Group, 2009) and Audiovisual Core (GBIF/TDWG Multimedia Resources Task Group, 2013) which help with this through a lightweight approach of specifying common field names and their definitions (such as “Capture Device”, “Taxon Coverage”, “Locality”, “Start Timestamp”). By using such standards, researchers can ensure that their metadata will be understood by others and be findable. It also enables a next generation of methods that could automatically generalise across multiple available data sets, since the metadata are compatible.

### (5) Data annotation

**Fig. 3:**
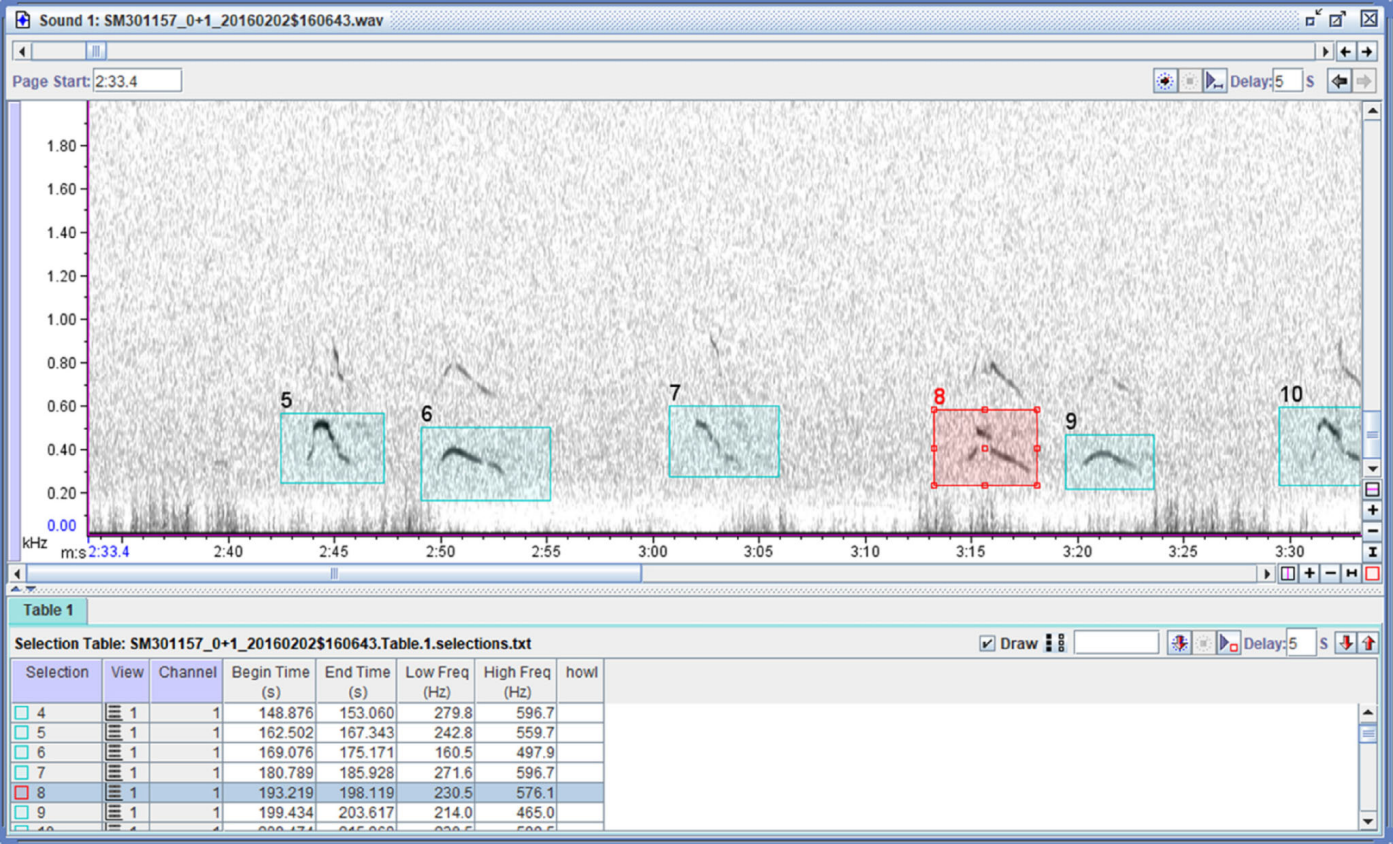
Example annotation of acoustic signals, in this case, wolf howls. Taken from Kershenbaum *et al*. (2019), showing a spectrogram generated using Raven Pro.

A well‐annotated data set is critical to the performance of a ML‐based automated detector (Fig. 3). When creating annotations, many decisions must be made, including which software will be used, the specific approach, as well as (often subjective) decisions regarding specifics about the granularity, or what “counts” as an annotation, for example individual vocalisation bouts or whole sequences. There have been calls to standardise annotation approaches in bioacoustics (Nicholson, 2023), similar to what has been done for human speech (Gibbon, Moore & Winski, 1998) and music (Humphrey *et al*., 2014). However, to our knowledge a standardised protocol does not yet exist, perhaps due to the diversity of signal types and research questions across bioacoustics and/or a lack of communication among fields. Here, we aim to provide some guidance for annotating a data set for automatic detection (Fig. 3).

Due to the relatively large amount of human investment required to get high‐quality annotations, researchers often ask themselves how many annotations are needed. This generally depends on the research question, and it is often recommended to annotate as many signals as possible, however there are more specific questions that can help guide these decisions. The first concerns the classes or discrete types of signals in your data set. For example, will you annotate every bird species in a long‐term recording? Will you annotate a single call type from a single species? Or will you annotate all the notes or elements in a sequence from a single individual? In addition, one must decide whether to annotate the “negative class” (often the “noise” or “absence” category). If doing exhaustive annotation where all the signals of interest are annotated, then it can be assumed that anything that is not annotated is the “negative class”. However, strategically annotating other “distractor/noise” sound events may improve detector performance, especially sounds occurring within the target frequency range which are loud or easily confused with the target signal. These “noise” labels can help with error analysis and with the training of an algorithm.

Decisions about the temporal scale of the annotations must also be made. A common approach is to annotate the smallest acoustic unit, for example note or syllable (Kershenbaum *et al*., 2016*a*); however, this method can be very time‐consuming for large data sets. For vocal sequences that are comprised of multiple acoustic units (e.g. gibbon vocalisations) another approach is to annotate particular call types or phrases within the longer sequence, e.g. annotate only the female gibbon contribution to the duet (Clink *et al*., 2023).

The number of annotations needed will be influenced by the research question and the choice of the automatic detection approach (see Section V.6) but may also be limited by external factors such as funding support for analysts. It is important to consider the diversity of signal types as well as background noise, and to work to include a distribution of annotations or samples across sites, times of day, groups, individuals, etc. A higher number of annotations (and therefore more available samples for training data) will likely improve detector performance and may be necessary in cases where the signals of interest are highly variable. In some cases, such as the use of transfer learning, a smaller number of training samples (~ 25) may be sufficient (Dufourq *et al*., 2022), but even in these cases, only a test set on the order of 100 examples would enable a reliable evaluation of the model. Researchers also need to make decisions about which target signals to include in their annotations, such as whether to include low SNR acoustic signals, signals that substantially overlap with non‐target signals, or signals that are abnormal in structure.

**Fig. 4:**
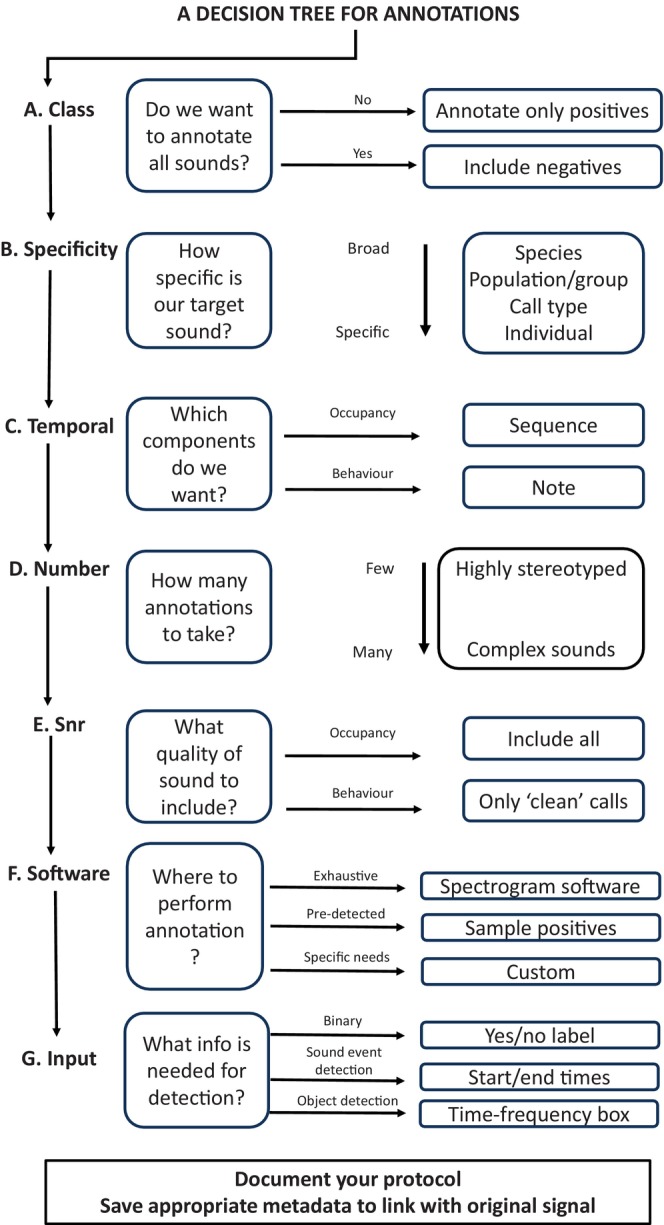
A flowchart for designing research questions in relation to automatic detection.

A common way to do annotations is by visualising spectrograms in a graphical user interface (GUI) such as Raven Pro (K. Lisa Yang Center for Conservation Bioacoustics, 2014), Sonic Visualizer (Cannam, Landone & Sandler, 2010) or Praat (Boersma & Weenink, 2007) and creating bounding boxes around the signal(s) of interest. Other possibilities include the use of an energy or coherence detector (Wijers *et al*., 2021) to identify all signals above a certain threshold in a given frequency range and then labelling these detections, applying an unsupervised clustering algorithm and labelling the batches of samples that have been grouped together, or the use of DL approaches to identify the start and stop times of signals of interest automatically, for example TweetyNet (Cohen *et al*., 2022). However, one must be cautious about mass semi‐automated annotations, since these may introduce non‐obvious bias that can affect the conclusions of the study. We recommend including random sampled manual inspection steps in the procedure. It is important to document your annotation protocol, including the decisions you made and why you made them, in a way that can be reproduced by others. We suggest including these protocols as online supporting information in publications. In addition, it is crucial to check both intra‐ and inter‐observer reliability for creating annotations (Duc *et al*., 2021). Figure 4 illustrates the considerations when choosing annotations.

### (6) Choose your detection pipeline

**Fig. 5:**
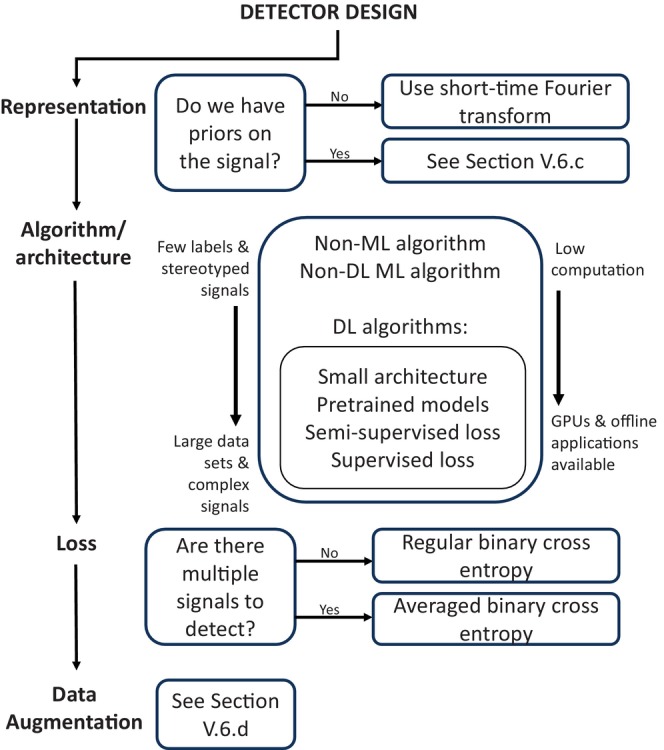
A flowchart showing the decisions necessary in automated detector design. DL, deep learning; GPU, graphical processing unit; ML, machine learning.

Figure 5 illustrates the considerations in designing and using different detector types.

#### (a) Interfacing with your pipeline

Selecting an automatic detection approach depends on factors such as technical familiarity, desired granularity, and budgetary constraints. Products such as Kaleidoscope (https://www.wildlifeacoustics.com/), PAMGuard (https://www.pamguard.org), and Arbimon (https://rfcx.org/ecoacoustics) provide easy‐to‐use interfaces for systems that can perform automatic detection on audio samples originating from a wide variety of environmental samples. These tools come equipped with traditional approaches rooted in standard signal‐processing techniques but are limited in their ability to utilise modern advances in ML. Conversely, modern DL frameworks, such as TensorFlow, PyTorch, and Keras (Stowell, 2022), as well as the models built with them, rarely come with an easy‐to‐use interface which makes them less accessible. Commercial approaches offering cloud‐based ML as a service (MLaaS) solutions, such as those from Amazon, IBM, or Microsoft, allow easier access to these advanced methods, but can be prohibitively expensive. Practitioners must decide whether easy‐to‐use tools are sufficient for the problem at hand, or whether it would be advantageous to exploit the often‐superior performance of DL methods, which require more investment of time, money or both. The complexity of the research question has a significant influence on the selection but may be outweighed by the need to invest further in expertise or funding.

In the case of any automatic detection approach, the pipeline must be evaluated in the context of the research questions which necessitates dividing the data properly to evaluate performance and generalisability, the choice of an appropriate detection mechanism, and the selection of relevant, comparable, and appropriate metrics.

#### (b) Split your data

As for most ML tasks, data sets should be split into “training”, “validation” and “test” subsets to ensure the true generalisability and comparability of a model's performance. The training set is directly used to optimise the detection algorithm, that is to learn from data; the validation set is to check performance during the training phase; the test set is to take a final estimate of the algorithm's performance on data it has never before encountered. This way, an amount of data (usually around 10–20% of the total data set) need to be kept unseen during training and validation of the model. This helps to avoid model overfitting, which would cause the model to learn only the characteristics of the training data, without the ability to generalise to new data, and would bias performance scores (James *et al*., 2013, p. 176).

The validation (or development) set is also used for selecting good values for parameters that affect the model but are not in the set of parameters it learns automatically (e.g. the number of layers) – this is so‐called “hyperparameter tuning”. This is especially useful in the case of DL models which involve empirical testing to determine the optimal configuration for elements such as optimisers, learning rates, or early stopping. The best‐performing model, as determined using the validation set, is then applied to the test set. The test set should not be used to fit the values of such hyperparameters or to compare model architectures since it would no longer serve for generalisation assessment; it is kept for final performance evaluation. Creating an effective test data set may include the selection of a separate microphone entry, specific time frames, separate recording locations, or subsets of vocalisations from an individual which were not included in the training set, amongst others. The general idea here is to separate the prediction capabilities of the computer model from recording specificities and data‐related biases. We always want to ensure that an automatic detection model is generalisable rather than specifically trained for a single recording setup, location, or individual.

To provide an example, in the case of creating a presence/absence detection model, one should not use annotations from the same file for training and testing. Instead, certain audio files should be used to create the training data set, and independent files should be used to test the detection model. Furthermore, the model should be applied to entire testing audio files and not only to parts of the test file that have been annotated, as this might result in an overly optimistic evaluation of the model and potential false positives would be missed.

#### (c) Pick your feature representation

Depending on the automatic detection approach, acoustic data may be transformed through feature extraction to ease the automatic detection process. In the computational bioacoustics literature, an array of such feature‐extraction methods can be found, each presenting their own advantages and limitations.

In bioacoustics, the dominant approach is undoubtedly spectral representations such as spectrograms or mel‐spectrograms. This type of representation usually allows for interpretable visualisation of acoustic data and provides an easy route to use popular vision‐based models such as CNNs for object detection and image classification. Despite this, some information from the raw waveform may get lost when computing these representations. This is especially the case for transient signals such as odontocetes' clicks which are poorly represented by Fourier transforms (Jiang *et al*., 2018). CNNs developed for spectrograms cannot be used directly for waveforms, because the data are of different dimensionality; however there have been a lot of recent developments in DL methods applied directly to waveforms and so this is increasingly feasible (Baevski *et al*., 2020).

DL methods now allow for high‐dimensional inputs such as whole spectrograms, with the succession of layers extracting higher level features and information. However, historically, users were the ones responsible for selecting relevant features to represent signals. In this context, low‐dimensional spectral summary statistics were often used, and given to a classification algorithm such as a support vector machine (Mitrovic, Zeppelzauer & Breiteneder, 2006). For relatively simple use cases, for example stereotyped signals and low background noise, this approach might suffice in obtaining satisfactory performances.

Recently, as stated in Section IV.1.a, extracting pretrained latent representations as features is also being adopted as a promising solution. This approach may imply additional effort on the part of the user and raises an array of questions on pretraining data sets, selected model architectures or the need for higher computational power. It can also prove successful in easing the downstream learning process or allowing for smaller annotated data sets in few‐shot learning perspectives.

Despite the advantage of using such abstract representations, using traditional engineered features such as fundamental frequency, call duration or number of notes may still prove to be effective depending on the task at hand. These can also be combined with features extracted from the time domain such as energy and zero‐crossing rates. These can then allow for the use of simpler algorithms which may be easier to implement and require little computational power and training time.

Overall, there is no such thing as the perfect feature‐extraction method for bioacoustics. Comparing different feature representations should always be the preferred approach and can be carried out on the previously mentioned validation set, ideally in a pilot study.

#### (d) Decide on feature transformation

Prior to feature extraction, specifically in the case of noisy recordings characterised by low SNR, some detectors may benefit from denoising, that is the automatic removal of background noise from the acoustic signal of interest. An extensive overview of recent approaches can be found in Xie, Colonna & Zhang (2021) with accessible open‐source solutions. Some of these methods are built on light‐weight algorithms such as spectral‐gating (Sainburg *et al*., 2020), others involve the use of DL with CNNs, Noise‐2‐Noise‐based approaches (Bergler *et al*., 2020), or denoising‐autoencoder models (Vickers *et al*., 2021; Yang *et al*., 2021).

Although it is useful in some applications, this pre‐processing step is not always recommended and must be used with caution as it may result in a loss of information. In some cases, noise can also be directly handled by the detector itself, especially when using noise‐resilient DL architectures or when stationary noise is not overlapping the target signals. In cases where noise reduction is applied prior to training, the evaluation and test data sets will need to be put through the same process, to ensure that training and testing data have comparable characteristics and contain similar acoustic information. When building a noise resilient model, one may also resort to multi‐condition training approaches. This method can simply adding noisy corrupted versions of the data to the training set or including both the original and the denoised versions of the data during training to help with model robustness to noisy acoustic contexts. This approach is fairly common in speech processing (Yin *et al*., 2015) but needs further exploration in bioacoustics.

Depending on the amount of training data available, data augmentation techniques may be used to artificially increase the variability of the data on which models are optimised. The choice of which augmentation technique to use depends on the application. One should aim to apply transformations that cover the range of variations found in real signals. However, care must be taken to avoid transformations that could invalidate the annotations. For instance, in a bird call detector, reversing sounds could be a tempting simple transformation, yet this could result in artificially making a bird call more similar to that of another species. Simple transformations may also create artefacts that can complicate the modelling, for example a pitch shift of a howl may also unrealistically shift the background noise.

Commonly used techniques include stretching or compressing the duration of acoustic signals, shifting their pitch, making small volume modifications, or adding a variety of noise or mixing with other audio events *via* some linear or non‐linear combination (e.g. taking one presence event and mixing it with one or more absence events). These transformations may also be combined to produce more variation. Going even further to generate training data, recently generative DL methods, such as Generative Adversarial Networks (GANs) have been proposed to generate synthetic examples (Wang, She & Ward, 2021; Bergler *et al*., 2022*a*).

#### (e) Decide on a method

##### (i) Deep learning or not

As mentioned above, the choice of a detection mechanism is dependent at least partially on the complexity of the problem. If the signals are well defined, have high SNR, are highly stereotyped, and the research question involves simple segmentation and can be done offline, a package such as Kaleidoscope or Arbimon may be more than adequate.

Using ML or DL may be advantageous in situations requiring a more complex analysis, such as call type classification, or where robustness to environmental noise is necessary (Aodha *et al*., 2018; Stowell, 2022). However, in situations where access to either a large amount of computing resources or the training/expertise to use them effectively is limited, the use of DL may not be possible. Additionally, it must be considered where the detection mechanism will be deployed. If access to a large computing cluster is readily available but the end result must function on a small device for field deployment, then a large and complex model may not work. Conversely, if the final model will only be used offline using minimal computing resources (budget GPU), then the model choice becomes somewhat more flexible. Different ML approaches are given in Table 1, together with their requirements and example studies.

**Table 1:** Different types of machine learning techniques.

Learning type	Labelled data requirements	Metrics	Visualisations	Examples
Supervised (segmentation, classification)	Large amount of labelled data	Accuracy, precision, recall, F‐score, AUROC, mAP, UAR	Confusion matrix ROC‐curve, PR‐curve	Bergler *et al*. (2022*b*)
Unsupervised or self‐supervised (clustering)	Labelled data not necessary	Reconstruction loss (MAE, MSE), homogeneity, completeness	Reconstructions, dim‐reduction (t‐SNE, UMAP)	Cuevas *et al*. (2017)
Semi‐supervised learning	Some labelled data – large amount of unlabelled data (optional)	Both supervised and unsupervised	Both supervised and unsupervised	Bermant *et al*. (2019); Saeed *et al*. (2021); Leroux *et al*. (2021); Hagiwara *et al*. (2022)

AUROC, area under the receiver operating characteristic curve; MAE, mean absolute error; mAP, mean average precision; MSE, mean squared error; PR, precision‐recall; ROC, receiver operating characteristic; t‐SNE, t‐stochastic neigbour embedding; UAR, unweighted average recall; UMAP, uniform manifold approximation and projection.

##### (ii) Choose your evaluation metrics

The evaluation of the automatic detection mechanism depends primarily on the type of task to be performed. A fully supervised detection/classification task is typically evaluated using metrics such as accuracy, precision, recall, F‐score, or area under the receiver operating characteristic curve (AUROC) (Lever, Krzywinski & Altman, 2016). These all provide different insights and can help evaluate how the model is performing. For example, precision indicates the fraction of relevant results (true positives) that are found among all detected events, whereas recall indicates the fraction of signals in the data set that were effectively found. Typically, a balance must be decided as to which metrics are most important for a particular task. For example, recall may be an important score to consider when detecting rare phenomena where missing a single detection of an underrepresented class may prove costly. Wrong choice of metrics may bias the results, for example, in the case of highly unbalanced data sets, that is when the acoustic object to be detected is rather underrepresented in the data set compared to negative labels, accuracy may be very high despite low performances on the small number of positive test samples.

Visualising results from supervised training methods can involve a confusion matrix, which is a table that shows the ground truth values on one axis and predicted values on the other, allowing easy‐to‐digest visual analysis of model performance. Another option is the receiver operating characteristic curve (ROC curve), which plots the trade‐off between true positive rate (TPR) and false positive rate (FPR) at all confidence thresholds, enabling the analyst to choose more easily a prediction threshold that suits their needs. The AUROC gives a summary of the model's performance across thresholds and is agnostic of threshold choice.

A similar visualisation to the ROC curve is the precision‐recall (PR) curve, which also highlights the balance between missing out events (false negatives) and making false alarms (false positives). The area under the PR curve is commonly referred to as the mean average precision (mAP). The important difference between PR and ROC curves is that the precision gives the proportion of correct detection among all detections and the FPR indicates the proportion of wrong detections among all negative examples. In the case of highly unbalanced data sets (e.g. 1% of positive examples), the FPR can be rather optimistic as compared to the precision, and thus the mAP might come out to be significantly lower than the AUROC. Detailed discussions on possible performance metrics can be found in Davis & Goadrich (2006) and Hildebrand *et al*. (2022).

Useful metrics for unsupervised learning are harder to identify, as they depend on the research question. If labelled data are available, they can be used to assess the quality of a clustering attempt by measuring completeness (across how many clusters are samples with the same label) or homogeneity (the proportion of samples in a cluster with the same label). Visualisation for unsupervised clustering results are often done by reducing the dimensionality to either two or three dimensions using t‐stochastic neighbour embedding (t‐SNE) (Maaten & Hinton, 2008), uniform manifold approximation and projection (UMAP) (McInnes, Healy & Melville, 2020), or a similar method.

### (7) Verifications – check your results

The verification of model performance on the test data should involve quantitative and qualitative evaluations. Quantitative metrics give the performance in terms of comparable values like accuracy, precision, or recall, or composite metrics like the F1 score, which combines both precision and recall, whilst qualitative metrics would help to understand the practical implications of the model. Qualitative analysis involves manually checking or visualising the predictions. This may involve plotting automatic segmentation results on spectrograms to account visually for the precision of detected time frames. It may also be carried out through a simple manual inspection of a subset of results. Careful manual analysis of the signals with missed detections or false alarms could help to identify the characteristics that trigger or do not trigger the models and help to improve the models further by adding the specific variations needed in the training data or in cleaning training data (especially wrong annotations or mislabelled data).

#### (a) When is a model good enough? Performance thresholds

Understanding the performance thresholds and being realistic about the task is a pragmatic way of approaching the problem. It is important to understand that ML models are statistical in nature and may never provide 100% performance even with perfect data or models. Understanding the limitations of the model and the desirable performance in the real‐world scenario can help set the thresholds for performance, for example, in a trade‐off between false positives and missed detections (Karnan, Akila & Krishnaraj, 2011). In some scenarios it may not be even practically feasible to achieve a desirable performance due to factors like overlapping sounds, environmental noise or very low SNR. But understanding and defining the problem based on a trade‐off between what is feasible with the acoustic data and what is desirable (for example, defining the range of distance within which the target species needs to be detected) could help define performance thresholds and build practical models.

#### (b) *How harmful are mistakes (false positives* versus *false negatives)?*

The use case for automatic detection will influence how much and what kind of errors are acceptable. For instance, for an analysis on vocal behaviour, missing a call in a sequence might strongly distort the results. Conversely, if occupancy trends are aimed for, missing one call in a sequence is insignificant, and imperfect detection can be incorporated into occupancy models, albeit as a naïve proxy (Bailey, MacKenzie & Nichols, 2014). Recall is thus more or less important depending on the type of study being conducted.

In general, false positives are undesirable, but a certain number might be acceptable (Shiu *et al*., 2020). In any case, converting the precision into the number of false positives per hour allows an unambiguous interpretation by the user and the planning of how to deal with false alarms.

Additionally, prior knowledge of vocal behaviour, such as sequence regularities, might allow filtering out of false positives. Such priors can be used to reduce confidence thresholds and increase the recall, but with the risk of imposing too strong priors and missing out on uncommon sequences.

#### (c) Reproducibility and accessibility

We also expect automated vocalisation detection systems to be made available to other users, thus broadening the contribution to the field of bioacoustics (especially to users without a strong computer science background). For this purpose, code for detection systems should be shared in comprehensive and accessible ways, such as in public repositories, and should be well documented with detailed user manuals (Braga *et al*., 2023). An easy way to make a detection model available to the community is to follow common standards for data input/output that will allow their integration into pre‐existing interfaces, such as ARISE (Hogeweg & Stowell, 2023) or Raven Pro (K. Lisa Yang Center for Conservation Bioacoustics, 2014).

Besides publishing code for experiments to be reproducible, data sets used for training and testing should be made available to the community for building new systems and comparing them using standard annotation protocols (see Section V.5). Indeed, public benchmarking data sets exist (Joly *et al*., 2015; Politis *et al*., 2021) but cover only a relatively small set of species targeted by bioacoustic studies.

#### (d) Access to raw recordings

In addition to the labelled training data set, raw recordings (as opposed to cut‐out snapshots) are of potential value to the research community, for example to train self‐supervised models, or for reuse in a search for other sounds/species. However, it might not always be feasible to make this readily accessible in public repositories due to storage and other constraints. We encourage researchers to store the raw recordings locally and share them on demand with the community or with interested parties.

## VI. WAYS FORWARD

We now consider some important ways forward for automatic detection for bioacoustics, including the challenges still to be overcome, best practices that should be implemented now, and the future directions of the field.

### (1) Challenges

#### (a) Bioacoustic challenges

Although automatic detection has already brought large improvements to the field of bioacoustics, challenges remain that are closely related to the nature of animal sound and/or the desired uses of such data. For instance, since population density estimates rely on detections, overestimations are possible from double‐counting individual vocalisations when they are picked up by multiple devices (Kimura *et al*., 2010; Marin‐Cudraz *et al*., 2019). The estimates can be further improved, and double‐counting issues can be reduced if calls can be localised and attributed to an identified individual (Nijman, 2007; Knight & Bayne, 2019; Hedley *et al*., 2021; Law *et al*., 2021).

Moreover, in most cases population density cannot be estimated without knowing the detection range of the system (Metcalf *et al*., 2023). The detection range of the acoustic signal will depend on multiple factors including source level and frequency range of the signal, characteristics of the habitat including ambient noise levels, vegetation and topography, along with the specifications of the ARU (Haupert, Sèbe & Sueur, 2022). However, detection range is often difficult to estimate, especially in forest environments or areas with extreme topography, and in many cases is ignored or assumed to be consistent across studies, when this may not be the case. When species of interest are near the limit of the detection range of the device, recordings of vocal signals may become attenuated or missed. This might cause problems in tasks that try to capture specific aspects of the vocalisation, for example to infer behaviour, caller identity, or communication patterns, rather than generic tasks looking at occupancy (Spillmann *et al*., 2017).

Even when accurately focusing on the vocal signals of a target species, animals might engage in simultaneous vocalisations or choruses (Torti *et al*., 2018), which makes a simple timestamped detection system insufficient for acoustic behaviour analysis. Also, it can be difficult to distinguish vocalisations of similar species if they share characteristics, for example dog barks and coyote barks share a number of similarities which make it difficult to determine which species produced the rapid‐fire sequence of noisy barks, although there are some quantitative differences (Feddersen‐Petersen, 2000).

#### (b) Computational challenges

Computational challenges in this field include questions of algorithms, data sets, computational efficiency, and computing platforms. One overarching challenge within ML in the broad sense, and with particular relevance to automatic detection, is the ability to generalise. For example, a model well‐trained for a particular species can perform poorly with even slight variations in recording devices, ambient noise, or operating environments. This could lead to low accuracy without further testing and adjustment. Creating scalable models that have the flexibility to add new species to the training data set, to increase the number of vocally active species that can be detected, is still a challenging task. Transferring knowledge from models built with data from one species to a new species without further training data is even more desirable. We also note that many models are highly task specific – the data specification, annotations, model architectures, and systems are highly optimised for best performance. For example, a system used to determine the occupancy of a species may not be suitable for individual identification, understanding communication, or behaviour patterns which superficially appear to be related but are subtly different tasks. It is not immediately clear to a user how far to trust in the generalisation of a detector.

Acquiring generic data sets that can address multiple tasks, such as population density estimation and behavioural characteristics, poses a significant challenge due to limitations in data collection strategies. Typically, data collection is initially planned to address specific tasks, which makes it difficult to acquire data sets that can be scaled to any given task. This is a challenge as it is essential to streamline and optimise the recordings to collect only data of interest to a particular task to increase storage and computational efficiency. But, at the same time, the data collected might not include the context or information needed to use it for a new task. A lack of generic, benchmark data sets has significant implications for the standardisation of methods in the field and the appropriate evaluation of research.

In bioacoustics, as in other fields, DL comes with very limited interpretability, an issue known as the “black box problem”. This amplifies the problem that conclusions drawn about DL models will be specific to the data set they were tested on, which significantly hinders the process of finding a consensus for the best architecture or training procedure to be used. In certain cases, it is also unclear how different research studies split their data sets and conduct model evaluation. Currently, little to no standards on the best approaches exist and without these best practices in place, authors will implement their own approaches within their research. The best opportunity to overcome issues such as these is firstly to encourage further development of public access or benchmark data sets, and secondly to probe models on their detailed behaviour regarding these data sets (Alain & Bengio, 2018). Within the current literature, the approach that authors have taken to implement their ML testing methodologies and model evaluation differs drastically. In most cases, comparisons are not made to existing results on data sets that are publicly available, instead, most studies present their findings related to their proposed method on the data set that was collected for the study. These observations are quite different to what has been observed within the computer vision and natural language processing literature where most studies compare their proposed method to various baselines and existing state‐of‐the‐art methods on the same data sets. Consequently, a comparison among research studies within bioacoustics is not feasible and determining the state‐of‐the‐art is non‐trivial. Various initiatives exist that provide bioacoustic benchmark data sets and standardised public evaluations, including automatic detection in particular, although these are neither as large nor as widely used as in mainstream ML application domains (Stowell *et al*., 2019; Ferrari *et al*., 2020; Hagiwara *et al*., 2022).

Training ML models, particularly deep neural networks, is computationally intensive. Specifically, computers, workstations, or servers with a large amount of processing power and GPU may be needed, to speed up the training or just to make it achievable in reasonable time. Furthermore, certain deep neural networks require a large amount of GPU RAM to load the model into memory, given the large number of trainable neural network parameters that need optimisation. The issue of access to computational power can exacerbate inequalities between people, institutions, and countries. However, the good news is that the widespread use of pretrained models can massively decrease the amount of computation needed: most researchers should not need to train a model from scratch. This helps to reduce inequalities as well as the carbon footprint incurred through a move to ML methods.

In conjunction with computation, data storage requirements have skyrocketed, with the amount of data being collected from PAM and necessities to store, share and create backups of these very large data sets. In certain cases, practitioners have had to ship hard drives physically across the world to share acoustic data sets, and in other cases practitioners share large data sets *via* cloud‐based solutions. It is unlikely that storing all audio for all projects is feasible, and yet discarding audio takes away the possibility of reanalysis or new uses. Bioacoustics will benefit from the development of mixed schemes with well‐designed heuristics to store some audio in full resolution (e.g. detected audio clips) and the remainder in highly compressed formats which are still reusable (e.g. embeddings or low‐bitrate lossy compression).

There are other considerations that arise from the large data volumes that are required both for training automatic detection systems, and for investigating biological questions using bioacoustics. Logistical challenges in maintaining the data collection devices include changing batteries, calibration of microphones, and general wear and tear. Sometimes the devices need to be deployed in remote, difficult‐to‐access, or even dangerous locations, which makes maintenance even more challenging. Therefore, the effort required to gather the volume of data needed for training automatic detection models needs to be considered carefully. However, artificial intelligence being a rapidly evolving field means that new techniques and models may ease (or indeed exacerbate) the problems of providing enough data.

### (2) Future directions

#### (a) Accessibility

The extent to which automatic detection for bioacoustics is accessible to a wide range of researchers across different fields and geographical regions is patchy and insufficient. Future developments in the field must include increasing the ease with which researchers can implement and customise the technology. Usable, stable, and open‐source tool kits with an associated GUI, and potentially a cloud‐based solution, can aid the entry of practitioners from a non‐ML background and reduce the learning curve. Standards‐based interoperability and component‐based approaches will help ensure that solutions remain well‐maintained and usable.

To move to the next generation of automatic detection, we look forward to further work developing the scale, reliability, and generality of ML methods in bioacoustics. But even considering the current state of the art, the barrier to entry for practitioners, students and researchers who are new to the field of ML is high (Broll & Whitaker, 2017; Schultze, Gruenefeld & Boll, 2020). This barrier is potentially even higher for newcomers in ML for bioacoustics than those entering the field of ML for computer vision or natural language processing. For the latter two, there are large quantities of educational material, including blog posts, online tutorials, books, videos, and software repositories. The number of research laboratories, and researchers from tertiary educational institutions working on automatic detection for PAM or bioacoustics in general is not evenly distributed between the Global North and South, and thus, the ability to train students may differ between regions. There is a pressing need for more educational material to become available so that those entering the field can rapidly learn the necessary skills to facilitate progress, and as such, we encourage researchers and practitioners to create and share open‐access educational material.

Complementary to educational materials is of course that systems themselves should be more accessible and user‐friendly. The required use of Python or R (let alone libraries such as Tensorflow, and repositories such as Github, etc.) acts as a barrier to many potential users, and so projects that develop good interfaces are to be celebrated. However, the pace of change in ML methods is rapid, as well as the diversity of platforms (e.g. mobile devices), so it is risky to advocate a single graphical interface. The solution is to rely on component‐based approaches and well‐documented standards; as long as user interfaces can use standards‐based methods to “talk to” algorithms and data sets, and each of these components can be replaced, substituted and improved, work in this domain will provide a good substrate that makes it easy for interface developers to add value to the work (Darwin Core Task Group, 2009; GBIF/TDWG Multimedia Resources Task Group, 2013). For all these components, the community needs to consider their maintenance models (open source or commercial, free or subscription based) and the ongoing maintenance of core components should not be left to chance.

#### (b) Foundation models

As with the maturation of ML in fields such as image or speech recognition, we expect animal vocalisation detection models progressively to standardise, not only in terms of model architectures but also in data representation. Indeed, pretrained models created from large data sets with a variety of species or taxa can yield rather generic embeddings, allowing good performances when fine‐tuning for a specific task, even when relatively few labels are available. Fields such as text processing and image recognition are beginning to move to a scale where “foundation models” emerge, meaning DL models which are trained across massive and highly varied data sets, whose scales lead to emergent generalisation behaviour and which can be reused for a wide range of downstream tasks (Bommasani *et al*., 2022). The same could happen for bioacoustics and automatic detection: although the size of the benefit is hard to foresee, large‐scale highly generalised models could indeed overcome the significant limitation in bioacoustics that many custom tasks do not come with strong training data sets. An alternative approach is few‐shot learning, recently explored to generalise robustly from as few as five examples (Nolasco *et al*., 2023). Such methods indicate that “one big data set” is not necessarily the main objective for the field. These trends may converge, with the many public bioacoustic data sets forming a richly structured pretraining curriculum for systems to generalise well from simple examples.

#### (c) Multi‐modal detection

Some challenges posed by automatic bioacoustic detection, including difficulties in separating individual emitters, precisely assessing population density, double counting, or missing detections, could potentially be eased by multi‐modal approaches: incorporating additional modalities such as images, video or GPS data into the automatic inference process. In fact, this may result in complementary information or context missing from the acoustic data and enhance the detector's performance, which can then enable uses such as abundance estimation (Akamatsu *et al*., 2013) and activity tracking (Li *et al*., 2020; Morrison & Novikova, 2023). Automatic multimodal approaches can also allow tackling complex and innovative behavioural questions for species known to communicate in multimodal ways, such as primates (Slocombe, Waller & Liebal, 2011; Liebal & Oña, 2018) and spiders (Uetz & Roberts, 2002; Hebets, 2005). Multimodal data thus presents many advantages for automatic bioacoustic detection, all the while raising an array of limitations and adding a certain degree of complexity to ML solutions. Recording multimodal data is a first important challenge which can be partly addressed through the increasing availability of new efficient hardware solutions, such as lightweight, inexpensive camera traps and drones. The automatic processing of non‐acoustic data is also being investigated and numerous ML models exist as promising solutions (Akamatsu *et al*., 2013). Yet, the simplicity, diversity and quantity of information contained in bioacoustic data seem to make it a superior solution in most detection tasks (Enari *et al*., 2019), at least until vision‐based ML and visual recording hardware/large data storage and processing show significant improvements.

#### (d) Biologists and computer scientists working together on the design loop

Some ML models and systems are designed without the full domain knowledge or context of the problem being addressed. There needs to be close collaboration between the ML engineer designing the systems and training models, and biological scientists, as domain experts, who can validate the solutions and performance of the models. The process pipeline needs to be designed such that domain experts closely monitor every stage from the methodology for data collection, design of data‐collection devices, data annotation techniques or methodology, data splits, model architecture (including inputs and outputs), and performance metrics and performance threshold values. It is also worth noting that the same biologists may also be the ideal audience for the commercialisation of foundational models once they become available and the technologies and methods are easily accessible. The system should be iteratively improved with active feedback from experts in the field or through the knowledge of the domain expert. This in turn maps to the process flow standardisation discussed in earlier sections.

Since bioacoustic tasks deal with big data sets, demanding high computational power, there needs to be consideration of the environmental impacts of data storage, data transfer, computation power in terms of model training, validation or deployment in the real world. Training ML models is computationally very expensive and the use of GPUs results in large amounts of energy consumption. This raises the question of sustainability with respect to the research being conducted. Various independent researchers training similar models on the same data sets would result in a suboptimal use of resources. Energy consumption may be reduced by training smaller models (by model pruning, or “distillation”) or by sharing models. There are options of cloud storage or cloud computations (Aide *et al*., 2013) which could benefit from the usage of green data centres in remote locations (Ministry of Local Government and Modernisation, 2021) that have green infrastructure for energy production (through renewable energy sources) and are perhaps less harmful to the environment, rather than local GPUs or server solutions.

It is also important to think of low‐footprint, low‐power‐usage models and systems in real‐world deployment for data collection or final deployment. Currently, many research studies are applying automatic detection algorithms on data that were collected in the past. We, however, anticipate that the field will move towards real‐time algorithms which require systems that consume less energy in comparison to modern GPUs. To achieve this, more efforts are required within model compression, for these models to be embedded into small devices during data collection or deployment in the field.

Automatic detection holds large opportunities for advances in the field of conservation and welfare, and drawing on the domain knowledge of biologists not currently involved in bioacoustics can open up new research directions. The advantages of processing large amounts of acoustic data seem clear to those currently involved in the field, but the wider biological community should be involved to find new fundamental research questions in the field of ecology and evolution (Clutton‐Brock & Sheldon, 2010; De Frenne *et al*., 2018), for example around species occurrence (Sebastián‐González *et al*., 2015; Rice *et al*., 2021; Sattar, 2023), population density (Marques *et al*., 2013) and diversity (Kotera & Phillott, 2022), habitat use (Brookes, Bailey & Thompson, 2013; Kotila *et al*., 2023), phenology (Dede *et al*., 2014; Monczak *et al*., 2017), and the early detection of invasive species (Juanes, 2018). Such questions offer opportunities for research into major conservation challenges like biodiversity loss and the effects of climate change (Sugai & Llusia, 2019; Ross *et al*., 2023). Presently, studies driven by existing bioacoustics practitioners mostly focus on occurrence, or spatial or temporal distribution of a single species, whereas the advancement of automatic detection potentially allows for a focus on multiple species and to map biodiversity and potentially the functioning of whole ecosystems (Ross *et al*., 2018).

Another example of how biologists and ecologists can steer the direction in which automatic detection may be developed in the future is to identify research questions without current technological solutions. For example, although detecting signs of poor animal welfare in captivity has been the subject of many studies (Zhang *et al*., 2022; Mao *et al*., 2022), there are comparably very few studies investigating the welfare of wild animals (Mcloughlin *et al*., 2019). This is surprising given the great potential acoustic monitoring of threatened species could provide, for example on species' reproduction or social behaviour (Teixeira, Maron & van Rensburg, 2019; Greggor *et al*., 2021).

## VII. CONCLUSIONS

(1) Automatic detection is no longer an optional capability in bioacoustics. Increasing data volumes, the need for near‐real‐time analysis, and the expanding range of questions that biologists want to answer using passive acoustics mean that opening up the capabilities of this promising technology requires parallel new developments in the field of machine learning (ML).
(2) Mature fields in ML, such as image or voice recognition, are not immediately transferrable to automatic detection in bioacoustics. Close cooperation between biologist practitioners and ML developers will help advance solution creation by (*a*) providing developers with an understanding of the problems facing bioacoustics practitioners, and (*b*) informing biologists as to what can and cannot be provided by the state of the art in ML.
(3) Despite the challenges, impressive advances in ML, particularly deep neural networks, hold out the potential for very significant developments that would cut processing time and enable a new wave of bioacoustics applications.
(4) Application development pipelines are of necessity problem specific, however, certain guidelines and workflows should smooth the integration of solutions constrained both by the biological features of the problem, and by the available ML capabilities.
(5) In summary, integrating multiple disciplines, leveraging new ML technology, and rigorous standardisation of protocols and data sets should open up multiple new opportunities for ecological and behavioural research through automated detection for bioacoustics.

## References

[brv13155-bib-0001] Abrahams, C. & Geary, M. (2020). Combining bioacoustics and occupancy modelling for improved monitoring of rare breeding bird populations. Ecological Indicators 112, 106131.

[brv13155-bib-0002] Acevedo, M. A. , Corrada‐Bravo, C. J. , Corrada‐Bravo, H. , Villanueva‐Rivera, L. J. & Aide, T. M. (2009). Automated classification of bird and amphibian calls using machine learning: a comparison of methods. Ecological Informatics 4, 206–214.

[brv13155-bib-0003] Aide, T. M. , Corrada‐Bravo, C. , Campos‐Cerqueira, M. , Milan, C. , Vega, G. & Alvarez, R. (2013). Real‐time bioacoustics monitoring and automated species identification. PeerJ 1, e103. 23882441 10.7717/peerj.103 PMC3719130

[brv13155-bib-0004] Akamatsu, T. , Ura, T. , Sugimatsu, H. , Bahl, R. , Behera, S. , Panda, S. , Khan, M. , Kar, S. K. , Kar, C. S. , Kimura, S. & Sasaki‐Yamamoto, Y. (2013). A multimodal detection model of dolphins to estimate abundance validated by field experiments. The Journal of the Acoustical Society of America 134, 2418–2426. 23968039 10.1121/1.4816554

[brv13155-bib-0005] Alain, G. & Bengio, Y. (2018). Understanding intermediate layers using linear classifier probes. arXiv. http://arxiv.org/abs/1610.01644. Accessed 5 July 2023.

[brv13155-bib-0006] Alcocer, I. , Lima, H. , Sugai, L. S. M. & Llusia, D. (2022). Acoustic indices as proxies for biodiversity: a meta‐analysis. Biological Reviews 97, 2209–2236. 35978471 10.1111/brv.12890 PMC9804652

[brv13155-bib-0007] Anderson, S. E. , Dave, A. S. & Margoliash, D. (1996). Template‐based automatic recognition of birdsong syllables from continuous recordings. The Journal of the Acoustical Society of America 100, 1209–1219. 8759970 10.1121/1.415968

[brv13155-bib-0008] Aodha, O. M. , Gibb, R. , Barlow, K. E. , Browning, E. , Firman, M. , Freeman, R. , Harder, B. , Kinsey, L. , Mead, G. R. , Newson, S. E. , Pandourski, I. , Parsons, S. , Russ, J. , Szodoray‐Paradi, A. , Szodoray‐Paradi, F. , *et al*. (2018). Bat detective—deep learning tools for bat acoustic signal detection. PLoS Computational Biology 14, e1005995. 29518076 10.1371/journal.pcbi.1005995 PMC5843167

[brv13155-bib-0009] Baevski, A. , Zhou, Y. , Mohamed, A. & Auli, M. (2020). wav2vec 2.0: a framework for self‐supervised learning of speech representations. Advances in Neural Information Processing Systems (NeurIPS) 33, 12449–12460.

[brv13155-bib-0010] Bailey, L. L. , MacKenzie, D. I. & Nichols, J. D. (2014). Advances and applications of occupancy models. Methods in Ecology and Evolution 5, 1269–1279.

[brv13155-bib-0011] Barker, D. J. , Herrera, C. & West, M. O. (2014). Automated detection of 50‐kHz ultrasonic vocalizations using template matching in XBAT. Journal of Neuroscience Methods 236, 68–75. 25128724 10.1016/j.jneumeth.2014.08.007 PMC4169788

[brv13155-bib-0012] Bee, M. A. (2012). Sound source perception in anuran amphibians. Current Opinion in Neurobiology 22, 301–310. 22265243 10.1016/j.conb.2011.12.014 PMC3338885

[brv13155-bib-0013] Bergler, C. , Barnhill, A. , Perrin, D. , Schmitt, M. , Maier, A. & Nöth, E. (2022 *a*). ORCA‐WHISPER: an automatic killer whale sound type generation toolkit using deep learning. In *Interspeech*, 2022 pp. 2413–2417. ISCA.

[brv13155-bib-0014] Bergler, C. , Schmitt, M. , Maier, A. , Smeele, S. , Barth, V. & Nöth, E. (2020). ORCA‐CLEAN: a deep Denoising toolkit for killer whale communication. In *Interspeech*, pp. 1136–1140. ISCA.

[brv13155-bib-0015] Bergler, C. , Smeele, S. Q. , Tyndel, S. A. , Barnhill, A. , Ortiz, S. T. , Kalan, A. K. , Cheng, R. X. , Brinkløv, S. , Osiecka, A. N. , Tougaard, J. , Jakobsen, F. , Wahlberg, M. , Nöth, E. , Maier, A. & Klump, B. C. (2022 *b*). ANIMAL‐SPOT enables animal‐independent signal detection and classification using deep learning. Scientific Reports 12, 21966. 36535999 10.1038/s41598-022-26429-y PMC9763499

[brv13155-bib-0016] Bermant, P. C. (2021). BioCPPNet: automatic bioacoustic source separation with deep neural networks. Scientific Reports 11, 23502. 34873197 10.1038/s41598-021-02790-2 PMC8648737

[brv13155-bib-0017] Bermant, P. C. , Bronstein, M. M. , Wood, R. J. , Gero, S. & Gruber, D. F. (2019). Deep machine learning techniques for the detection and classification of sperm whale bioacoustics. Scientific Reports 9, 12588. 31467331 10.1038/s41598-019-48909-4 PMC6715799

[brv13155-bib-0018] Best, P. , Ferrari, M. , Poupard, M. , Paris, S. , Marxer, R. , Symonds, H. , Spong, P. & Glotin, H. (2020). Deep learning and domain transfer for orca vocalization detection. In *2020 International Joint Conference on Neural Networks (IJCNN)*, pp. 1–7.

[brv13155-bib-0019] Best, P. , Marxer, R. , Paris, S. & Glotin, H. (2022). Temporal evolution of the Mediterranean fin whale song. Scientific Reports 12, 13565. 35945237 10.1038/s41598-022-15379-0 PMC9363496

[brv13155-bib-0020] Best, P. , Paris, S. , Glotin, H. & Marxer, R. (2023). Deep audio embeddings for vocalisation clustering. PLoS One 18, e0283396. 37428759 10.1371/journal.pone.0283396 PMC10332598

[brv13155-bib-0021] Bittle, M. & Duncan, A. (2013). A review of current marine mammal detection and classification algorithms for use in automated passive acoustic monitoring. In *Proceedings of Acoustics, Victor Harbor, Australia*.

[brv13155-bib-0022] Boakes, E. H. , McGowan, P. J. K. , Fuller, R. A. , Chang‐qing, D. , Clark, N. E. , O'Connor, K. & Mace, G. M. (2010). Distorted views of biodiversity: spatial and temporal bias in species occurrence data. PLoS Biology 8, e1000385. 20532234 10.1371/journal.pbio.1000385 PMC2879389

[brv13155-bib-0023] Boersma, P. & Weenink, D. (2007). PRAAT: doing phonetics by computer (version 5.3.51). https://github.com/praat/praat. Accessed 13 June 2024.

[brv13155-bib-0024] Bommasani, R. , Hudson, D. A. , Adeli, E. , Altman, R. , Arora, S. , von Arx, S. , Bernstein, M. S. , Bohg, J. , Bosselut, A. , Brunskill, E. , Brynjolfsson, E. , Buch, S. , Card, D. , Castellon, R. , Chatterji, N. , *et al*. (2022). On the opportunities and risks of foundation models. arXiv. http://arxiv.org/abs/2108.07258. Accessed 7 August 2023.

[brv13155-bib-0025] Bøttcher, A. , Gero, S. , Beedholm, K. , Whitehead, H. & Madsen, P. T. (2018). Variability of the inter‐pulse interval in sperm whale clicks with implications for size estimation and individual identification. The Journal of the Acoustical Society of America 144, 365–374. 30075661 10.1121/1.5047657

[brv13155-bib-0026] Bouchet, H. , Blois‐Heulin, C. & Lemasson, A. (2013). Social complexity parallels vocal complexity: a comparison of three non‐human primate species. Frontiers in Psychology 4, 390. 23847565 10.3389/fpsyg.2013.00390 PMC3705190

[brv13155-bib-0027] Braga, P. H. P. , Hébert, K. , Hudgins, E. J. , Scott, E. R. , Edwards, B. P. M. , Sánchez Reyes, L. L. , Grainger, M. J. , Foroughirad, V. , Hillemann, F. , Binley, A. D. , Brookson, C. B. , Gaynor, K. M. , Shafiei Sabet, S. , Güncan, A. , Weierbach, H. , *et al*. (2023). Not just for programmers: how GitHub can accelerate collaborative and reproducible research in ecology and evolution. Methods in Ecology and Evolution 14, 1364–1380.

[brv13155-bib-0028] British Trust For Ornithology (2023). BTO acoustic pipeline. BTO ‐ British Trust for Ornithology. https://www.bto.org/our‐science/products‐and‐technologies/bto‐acoustic‐pipeline. Accessed 13 June 2024.

[brv13155-bib-0029] Broll, B. & Whitaker, J. (2017). DeepForge: an open source, collaborative environment for reproducible deep learning. Open Review.

[brv13155-bib-0030] Brookes, K. L. , Bailey, H. & Thompson, P. M. (2013). Predictions from harbor porpoise habitat association models are confirmed by long‐term passive acoustic monitoring. The Journal of the Acoustical Society of America 134, 2523–2533. 23968050 10.1121/1.4816577

[brv13155-bib-0031] Browning, E. , Gibb, R. , Glover‐Kapfer, P. & Jones, K. E. (2017). Passive acoustic monitoring in ecology and conservation . Report, WWF‐UK.

[brv13155-bib-0032] Burgos, G. & Zuberogoitia, I. (2020). A telemetry study to discriminate between home range and territory size in tawny owls. Bioacoustics 29, 109–121.

[brv13155-bib-0033] Buxton, R. T. , McKenna, M. F. , Clapp, M. , Meyer, E. , Stabenau, E. , Angeloni, L. M. , Crooks, K. & Wittemyer, G. (2018). Efficacy of extracting indices from large‐scale acoustic recordings to monitor biodiversity. Conservation Biology 32, 1174–1184. 29676813 10.1111/cobi.13119

[brv13155-bib-0034] Cannam, C. , Landone, C. & Sandler, M. (2010). Sonic visualiser: an open source application for viewing, analysing, and annotating music audio files. In *Proceedings of the 18th ACM International Conference on Multimedia*, pp. 1467–1468. Association for Computing Machinery, New York, NY, USA.

[brv13155-bib-0035] Casaer, J. , Milotic, T. , Liefting, Y. , Desmet, P. & Jansen, P. (2019). Agouti: a platform for processing and archiving of camera trap images. Biodiversity Information Science and Standards 3, e46690.

[brv13155-bib-0036] Castellote, M. & Fossa, F. (2006). Measuring acoustic activity as a method to evaluate welfare in captive beluga whales (*Delphinapterus leucas*). Aquatic Mammals 32, 325–333.

[brv13155-bib-0037] Clark, F. E. & Dunn, J. C. (2022). From soundwave to soundscape: a guide to acoustic research in captive animal environments. Frontiers in Veterinary Science 9, 889117. 35782565 10.3389/fvets.2022.889117 PMC9244380

[brv13155-bib-0038] Clark, M. L. , Salas, L. , Baligar, S. , Quinn, C. A. , Snyder, R. L. , Leland, D. , Schackwitz, W. , Goetz, S. J. & Newsam, S. (2023). The effect of soundscape composition on bird vocalization classification in a citizen science biodiversity monitoring project. Ecological Informatics 75, 102065.

[brv13155-bib-0039] Clink, D. J. , Kier, I. , Ahmad, A. H. & Klinck, H. (2023). A workflow for the automated detection and classification of female gibbon calls from long‐term acoustic recordings. Frontiers in Ecology and Evolution 11, 1071640.

[brv13155-bib-0040] Clutton‐Brock, T. & Sheldon, B. C. (2010). Individuals and populations: the role of long‐term, individual‐based studies of animals in ecology and evolutionary biology. Trends in Ecology & Evolution 25, 562–573. 20828863 10.1016/j.tree.2010.08.002

[brv13155-bib-0041] Çoban, E. B. , Pir, D. , So, R. & Mandel, M. I. (2020). Transfer learning from Youtube soundtracks to tag arctic ecoacoustic recordings. In *ICASSP 2020–2020 IEEE International Conference on Acoustics, Speech and Signal Processing (ICASSP)*, pp. 726–730.

[brv13155-bib-0042] Coffey, K. R. , Marx, R. E. & Neumaier, J. F. (2019). DeepSqueak: a deep learning‐based system for detection and analysis of ultrasonic vocalizations. Neuropsychopharmacology 44, 859–868. 30610191 10.1038/s41386-018-0303-6 PMC6461910

[brv13155-bib-0043] Cohen, Y. , Nicholson, D. A. , Sanchioni, A. , Mallaber, E. K. , Skidanova, V. & Gardner, T. J. (2022). Automated annotation of birdsong with a neural network that segments spectrograms. eLife 11, e63853. 35050849 10.7554/eLife.63853 PMC8860439

[brv13155-bib-0044] Cole, J. S. , Michel, N. L. , Emerson, S. A. & Siegel, R. B. (2022). Automated bird sound classifications of long‐duration recordings produce occupancy model outputs similar to manually annotated data. Ornithological Applications 124, 2, duac003.

[brv13155-bib-0045] Coleman, L. S. , Ford, W. M. , Dobony, C. A. & Britzke, E. R. (2014). Comparison of radio‐telemetric home‐range analysis and acoustic detection for little brown bat habitat evaluation. Northeastern Naturalist 21, 431–445.

[brv13155-bib-0046] Cretois, B. , Rosten, C. M. & Sethi, S. S. (2022). Voice activity detection in eco‐acoustic data enables privacy protection and is a proxy for human disturbance. Methods in Ecology and Evolution 13, 2865–2874.

[brv13155-bib-0047] Cuevas, A. , Veragua, A. , Español‐Jiménez, S. , Chiang, G. & Tobar, F. (2017). Unsupervised blue whale call detection using multiple time‐frequency features. In *2017 CHILEAN Conference on Electrical, Electronics Engineering, Information and Communication Technologies (CHILECON)*, pp. 1–6.

[brv13155-bib-0048] Darwin Core Task Group (2009). Darwin Core. https://www.tdwg.org/standards/dwc/. Accessed 5 July 2023.

[brv13155-bib-0049] Davis, J. & Goadrich, M. (2006). The relationship between precision‐recall and ROC curves. In *Proceedings of the 23rd International Conference on Machine Learning*, pp. 233–240. Association for Computing Machinery, New York, NY, USA.

[brv13155-bib-0050] Dawson, D. K. & Efford, M. G. (2009). Bird population density estimated from acoustic signals. Journal of Applied Ecology 46, 1201–1209.

[brv13155-bib-0051] De Frenne, P. , Van Langenhove, L. , Van Driessche, A. , Bertrand, C. , Verheyen, K. & Vangansbeke, P. (2018). Using archived television video footage to quantify phenology responses to climate change. Methods in Ecology and Evolution 9, 1874–1882.

[brv13155-bib-0052] Dede, A. , Öztürk, A. A. , Akamatsu, T. , Tonay, A. M. & Öztürk, B. (2014). Long‐term passive acoustic monitoring revealed seasonal and diel patterns of cetacean presence in the Istanbul Strait. Journal of the Marine Biological Association of the United Kingdom 94, 1195–1202.

[brv13155-bib-0053] Denton, T. , Wisdom, S. & Hershey, J. R. (2021). Improving bird classification with unsupervised sound separation. arXiv. http://arxiv.org/abs/2110.03209. Accessed 6 July 2023.

[brv13155-bib-0054] Derrickson, K. C. (1988). Variation in repertoire presentation in northern mockingbirds. The Condor 90, 592–606.

[brv13155-bib-0055] Duan, S. , Zhang, J. , Roe, P. , Wimmer, J. , Dong, X. , Truskinger, A. & Towsey, M. (2013). Timed probabilistic automaton: a bridge between raven and Song scope for automatic species recognition. Proceedings of the AAAI Conference on Artificial Intelligence 27, 1519–1524.

[brv13155-bib-0056] Duc, P. N. H. , Torterotot, M. , Samaran, F. , White, P. R. , Gérard, O. , Adam, O. & Cazau, D. (2021). Assessing inter‐annotator agreement from collaborative annotation campaign in marine bioacoustics. Ecological Informatics 61, 101185.

[brv13155-bib-0057] Dufourq, E. , Batist, C. , Foquet, R. & Durbach, I. (2022). Passive acoustic monitoring of animal populations with transfer learning. Ecological Informatics 70, 101688.

[brv13155-bib-0058] Dufourq, E. , Durbach, I. , Hansford, J. P. , Hoepfner, A. , Ma, H. , Bryant, J. V. , Stender, C. S. , Li, W. , Liu, Z. , Chen, Q. , Zhou, Z. & Turvey, S. T. (2021). Automated detection of Hainan gibbon calls for passive acoustic monitoring. Remote Sensing in Ecology and Conservation 7, 475–487.

[brv13155-bib-0059] Dunn, J. C. & Smaers, J. B. (2018). Neural correlates of vocal repertoire in primates. Frontiers in Neuroscience 12, 534. 30140202 10.3389/fnins.2018.00534 PMC6095195

[brv13155-bib-0060] Enari, H. , Enari, H. S. , Okuda, K. , Maruyama, T. & Okuda, K. N. (2019). An evaluation of the efficiency of passive acoustic monitoring in detecting deer and primates in comparison with camera traps. Ecological Indicators 98, 753–762.

[brv13155-bib-0061] Erbe, C. & Thomas, J. A. (eds) (2022). Exploring Animal Behavior through Sound: Volume 1: Methods. Springer Nature, Gewerbestrasse, Switzerland.

[brv13155-bib-0062] Exadaktylos, V. , Silva, M. , Aerts, J.‐M. , Taylor, C. J. & Berckmans, D. (2008). Real‐time recognition of sick pig cough sounds. Computers and Electronics in Agriculture 63, 207–214.

[brv13155-bib-0063] Fairbrass, A. J. , Firman, M. , Williams, C. , Brostow, G. J. , Titheridge, H. & Jones, K. E. (2019). CityNet—deep learning tools for urban ecoacoustic assessment. Methods in Ecology and Evolution 10, 186–197.

[brv13155-bib-0064] Feddersen‐Petersen, D. U. (2000). Vocalization of European wolves (*Canis lupus lupus* L.) and various dog breeds (*Canis lupus* f. fam.). Archives Animal Breeding 43, 387–398.

[brv13155-bib-0065] Ferrari, M. , Glotin, H. , Marxer, R. & Asch, M. (2020). DOCC10: open access dataset of marine mammal transient studies and end‐to‐end CNN classification. In *2020 International Joint Conference on Neural Networks (IJCNN)*, pp. 1–8. IJCNN, Glasgow, UK.

[brv13155-bib-0066] Fleishman, E. , Cholewiak, D. , Gillespie, D. , Helble, T. , Klinck, H. , Nosal, E. M. & Roch, M. A. (2023). Ecological inferences about marine mammals from passive acoustic data. Biological Reviews 98, 1633–1647. 37142263 10.1111/brv.12969

[brv13155-bib-0067] Ford, J. K. B. (1991). Vocal traditions among resident killer whales (*Orcinus orca*) in coastal waters of British Columbia. Canadian Journal of Zoology 69, 1454–1483.

[brv13155-bib-0068] Fox, E. J. S. , Roberts, J. D. & Bennamoun, M. (2008). Call‐independent individual identification in birds. Bioacoustics 18, 51–67.

[brv13155-bib-0069] Frick, W. F. (2013). Acoustic monitoring of bats, considerations of options for long‐term monitoring. Therya 4, 69–70.

[brv13155-bib-0070] Frommolt, K.‐H. & Tauchert, K.‐H. (2014). Applying bioacoustic methods for long‐term monitoring of a nocturnal wetland bird. Ecological Informatics 21, 4–12.

[brv13155-bib-0071] Ganchev, T. D. (2020). Chapter 8 ‐ ubiquitous computing and biodiversity monitoring. In Advances in Ubiquitous Computing (ed. A. Neustein ), pp. 239–259. Elsevier Science, Amsertdam, Netherlands.

[brv13155-bib-0072] Garland, E. C. , Castellote, M. & Berchok, C. L. (2015). Beluga whale (*Delphinapterus leucas*) vocalizations and call classification from the eastern Beaufort Sea population. The Journal of the Acoustical Society of America 137, 3054–3067. 26093397 10.1121/1.4919338

[brv13155-bib-0073] Garland, E. C. , Goldizen, A. W. , Rekdahl, M. L. , Constantine, R. , Garrigue, C. , Hauser, N. D. , Poole, M. M. , Robbins, J. & Noad, M. J. (2011). Dynamic horizontal cultural transmission of humpback whale song at the ocean basin scale. Current Biology 21, 687–691. 21497089 10.1016/j.cub.2011.03.019

[brv13155-bib-0074] GBIF/TDWG Multimedia Resources Task Group (2013). Audiovisual Core Multimedia Resources Metadata Schema. https://www.tdwg.org/standards/ac/. Accessed 5 July 2023.

[brv13155-bib-0075] Gibb, R. , Browning, E. , Glover‐Kapfer, P. & Jones, K. E. (2019). Emerging opportunities and challenges for passive acoustics in ecological assessment and monitoring. Methods in Ecology and Evolution 10, 169–185.

[brv13155-bib-0076] Gibbon, D. , Moore, R. & Winski, R. (eds) (1998). Vol 1 Spoken Language System and Corpus Design. De Gruyter Mouton, Berlin, Boston.

[brv13155-bib-0077] Gillespie, D. & Chappell, O. (2002). An automatic system for detecting and classifying the vocalisations of harbour porpoises. Bioacoustics 13, 37–61.

[brv13155-bib-0078] Gillespie, D. , Mellinger, D. K. , Gordon, J. , McLaren, D. , Redmond, P. , McHugh, R. , Trinder, P. , Deng, X. Y. & Thode, A. (2009). PAMGUARD: Semiautomated, open source software for real‐time acoustic detection and localization of cetaceans. The Journal of the Acoustical Society of America, 125, 2547–2547.

[brv13155-bib-0079] Green, A. , Clark, C. , Favaro, L. , Lomax, S. & Reby, D. (2019). Vocal individuality of Holstein‐Friesian cattle is maintained across putatively positive and negative farming contexts. Scientific Reports 9, 18468. 31804583 10.1038/s41598-019-54968-4 PMC6895157

[brv13155-bib-0080] Greggor, A. L. , Masuda, B. , Gaudioso‐Levita, J. M. , Nelson, J. T. , White, T. H. , Shier, D. M. , Farabaugh, S. M. & Swaisgood, R. R. (2021). Pre‐release training, predator interactions and evidence for persistence of anti‐predator behavior in reintroduced 'alalā, Hawaiian crow. Global Ecology and Conservation 28, e01658.

[brv13155-bib-0081] Hagiwara, M. (2022). AVES: animal vocalization encoder based on self‐supervision. arXiv. http://arxiv.org/abs/2210.14493. Accessed 6 July 2023.

[brv13155-bib-0082] Hagiwara, M. , Hoffman, B. , Liu, J.‐Y. , Cusimano, M. , Effenberger, F. & Zacarian, K. (2022). BEANS: the benchmark of animal sounds. arXiv. http://arxiv.org/abs/2210.12300. Accessed 6 July 2023.

[brv13155-bib-0083] Hansen, P. (1979). Vocal learning: its role in adapting sound structures to long‐distance propagation, and a hypothesis on its evolution. Animal Behaviour 27, 1270–1271.

[brv13155-bib-0084] Harrington, F. H. & Mech, L. D. (1982). An analysis of howling response parameters useful for wolf pack censusing. The Journal of Wildlife Management 46, 686–693.

[brv13155-bib-0085] Haupert, S. , Sèbe, F. & Sueur, J. (2022). Physics‐based model to predict the acoustic detection distance of terrestrial autonomous recording units over the diel cycle and across seasons: insights from an Alpine and a Neotropical forest. Methods in Ecology and Evolution 14, 614–630.

[brv13155-bib-0086] Heaphy, K. & Cain, K. (2021). Song variation between sexes and among subspecies of New Zealand Fantail (*Rhipidura fuliginosa*). Emu ‐ Austral Ornithology 121, 198–210.

[brv13155-bib-0087] Heath, B. E. , Sethi, S. S. , Orme, C. D. L. , Ewers, R. M. & Picinali, L. (2021). How index selection, compression, and recording schedule impact the description of ecological soundscapes. Ecology and Evolution 11, 13206–13217. 34646463 10.1002/ece3.8042 PMC8495811

[brv13155-bib-0088] Hebets, E. A. (2005). Attention‐altering signal interactions in the multimodal courtship display of the wolf spider *Schizocosa uetzi* . Behavioral Ecology 16, 75–82. 10.1093/beheco/arn080 PMC2583108 19529816

[brv13155-bib-0089] Hebets, E. A. , Bern, M. , McGinley, R. H. , Roberts, A. , Kershenbaum, A. , Starrett, J. & Bond, J. E. (2021). Sister species diverge in modality‐specific courtship signal form and function. Ecology and Evolution 11, 852–871. 33520171 10.1002/ece3.7089 PMC7820158

[brv13155-bib-0090] Hedley, R. W. , Wilson, S. J. , Yip, D. A. , Li, K. & Bayne, E. M. (2021). Distance truncation via sound level for bioacoustic surveys in patchy habitat. Bioacoustics 30, 303–323.

[brv13155-bib-0091] Hendry, H. & Mann, C. (2018). Camelot—intuitive software for camera‐trap data management. Oryx 52, 15.

[brv13155-bib-0092] Hildebrand, J. A. , Frasier, K. E. , Helble, T. A. & Roch, M. A. (2022). Performance metrics for marine mammal signal detection and classification. The Journal of the Acoustical Society of America 151, 414–427. 35105012 10.1121/10.0009270

[brv13155-bib-0093] Hill, A. P. , Prince, P. , Snaddon, J. L. , Doncaster, C. P. & Rogers, A. (2019). AudioMoth: a low‐cost acoustic device for monitoring biodiversity and the environment. HardwareX 6, e00073.

[brv13155-bib-0094] Hogeweg, L. & Stowell, D. (2023). An API for AI species recognition. Arise. https://www.arise-biodiversity.nl/post/an-api-for-ai-species-recognition. Accessed 4 July 2023.

[brv13155-bib-0095] Hood, J. D. , Flogeras, D. G. & Theriault, J. A. (2016). Improved passive acoustic band‐limited energy detection for cetaceans. Applied Acoustics 106, 36–41.

[brv13155-bib-0096] Hsu, W.‐N. , Bolte, B. , Tsai, Y.‐H. H. , Lakhotia, K. , Salakhutdinov, R. & Mohamed, A. (2021). HuBERT: self‐supervised speech representation learning by masked prediction of hidden units. arXiv. http://arxiv.org/abs/2106.07447. Accessed 6 July 2023.

[brv13155-bib-0097] Humphrey, E. J. , Salamon, J. , Nieto, O. , Forsyth, J. , Bittner, R. M. & Bello, J. P. (2014). JAMS: a JSON annotated music specification for reproducible MIR research. In *ISMIR*, pp. 591–596.

[brv13155-bib-0098] James, G. , Witten, D. , Hastie, T. & Tibshirani, R. (2013). An Introduction to Statistical Learning: With Applications in R. Springer, New York, NY.

[brv13155-bib-0099] Jansson, A. , Humphrey, E. , Montecchio, N. , Bittner, R. , Kumar, A. & Weyde, T. (2017). Singing voice separation with deep U‐net convolutional networks. In *18th International Society for Music Information Retrieval Conference*, Suzhou, China. https://ismir2017.smcnus.org/. Accessed 6 July 2023.

[brv13155-bib-0100] Jiang, J. , Bu, L. , Wang, X. , Li, C. , Sun, Z. , Yan, H. , Hua, B. , Duan, F. & Yang, J. (2018). Clicks classification of sperm whale and long‐finned pilot whale based on continuous wavelet transform and artificial neural network. Applied Acoustics 141, 26–34.

[brv13155-bib-0101] Joly, A. , Goëau, H. , Glotin, H. , Spampinato, C. , Bonnet, P. , Vellinga, W.‐P. , Planqué, R. , Rauber, A. , Palazzo, S. , Fisher, B. & Müller, H. (2015). LifeCLEF 2015: multimedia life species identification challenges. In Experimental IR Meets Multilinguality, Multimodality, and Interaction (eds J. Mothe , J. Savoy , J. Kamps , K. Pinel‐Sauvagnat , G. Jones , E. San Juan , L. Capellato and N. Ferro ), pp. 462–483. Springer International Publishing, Cham.

[brv13155-bib-0102] Juanes, F. (2018). Visual and acoustic sensors for early detection of biological invasions: current uses and future potential. Journal for Nature Conservation 42, 7–11.

[brv13155-bib-0103] K. Lisa Yang Center for Conservation Bioacoustics (2014). Bioacoustics research program. https://ravensoundsoftware.com/. Accessed 5 July 2023.

[brv13155-bib-0104] Kahl, S. , Wood, C. M. , Eibl, M. & Klinck, H. (2021). BirdNET: a deep learning solution for avian diversity monitoring. Ecological Informatics 61, 101236.

[brv13155-bib-0105] Kalan, A. K. , Piel, A. K. , Mundry, R. , Wittig, R. M. , Boesch, C. & Kühl, H. S. (2016). Passive acoustic monitoring reveals group ranging and territory use: a case study of wild chimpanzees (*Pan troglodytes*). Frontiers in Zoology 13, 34. 27507999 10.1186/s12983-016-0167-8 PMC4977853

[brv13155-bib-0106] Karnan, M. , Akila, M. & Krishnaraj, N. (2011). Biometric personal authentication using keystroke dynamics: a review. Applied Soft Computing 11, 1565–1573.

[brv13155-bib-0107] Kershenbaum, A. , Blumstein, D. T. , Roch, M. A. , Akçay, Ç. , Backus, G. , Bee, M. A. , Bohn, K. , Cao, Y. , Carter, G. , Cäsar, C. , Coen, M. , DeRuiter, S. L. , Doyle, L. , Edelman, S. , Ferrer‐i‐Cancho, R. , *et al*. (2016 *a*). Acoustic sequences in non‐human animals: a tutorial review and prospectus. Biological Reviews 91, 13–52. 25428267 10.1111/brv.12160 PMC4444413

[brv13155-bib-0108] Kershenbaum, A. , Demartsev, V. , Gammon, D. E. , Geffen, E. , Gustison, M. L. , Ilany, A. & Lameira, A. R. (2021). Shannon entropy as a robust estimator of Zipf's Law in animal vocal communication repertoires. Methods in Ecology and Evolution 12, 553–564.

[brv13155-bib-0109] Kershenbaum, A. , Ilany, A. , Blaustein, L. & Geffen, E. (2012). Syntactic structure and geographical dialects in the songs of male rock hyraxes. Proceedings of the Royal Society B: Biological Sciences 279, 2974–2981. 10.1098/rspb.2012.0322 PMC3385477 22513862

[brv13155-bib-0110] Kershenbaum, A. , Owens, J. L. & Waller, S. (2019). Tracking cryptic animals using acoustic multilateration: a system for long‐range wolf detection. The Journal of the Acoustical Society of America 145, 1619–1628. 31067959 10.1121/1.5092973

[brv13155-bib-0111] Kershenbaum, A. & Roch, M. A. (2013). An image processing based paradigm for the extraction of tonal sounds in cetacean communications. The Journal of the Acoustical Society of America 134, 4435–4445. 25669255 10.1121/1.4828821 PMC3874055

[brv13155-bib-0112] Kershenbaum, A. , Root‐Gutteridge, H. , Habib, B. , Koler‐Matznick, J. , Mitchell, B. , Palacios, V. & Waller, S. (2016 *b*). Disentangling canid howls across multiple species and subspecies: structure in a complex communication channel. Behavioural Processes 124, 149–157. 26809021 10.1016/j.beproc.2016.01.006

[brv13155-bib-0113] Kershenbaum, A. , Sayigh, L. S. & Janik, V. M. (2013). The encoding of individual identity in dolphin signature whistles: how much information is needed? PLoS One 8, e77671. 24194893 10.1371/journal.pone.0077671 PMC3806847

[brv13155-bib-0114] Kimura, S. , Akamatsu, T. , Li, S. , Dong, S. , Dong, L. , Wang, K. , Wang, D. & Arai, N. (2010). Density estimation of Yangtze finless porpoises using passive acoustic sensors and automated click train detection. The Journal of the Acoustical Society of America 128, 1435. 20815477 10.1121/1.3442574

[brv13155-bib-0115] Knight, E. C. & Bayne, E. M. (2019). Classification threshold and training data affect the quality and utility of focal species data processed with automated audio‐recognition software. Bioacoustics 28, 539–554.

[brv13155-bib-0116] Kohlberg, A. B. , Myers, C. R. & Figueroa, L. L. (2024). From buzzes to bytes: a systematic review of automated bioacoustics models used to detect, classify and monitor insects. Journal of Applied Ecology 61, 1199–1211.

[brv13155-bib-0117] Kotera, M. M. & Phillott, A. D. (2022). Calls for conservation: a review of bioacoustics monitoring with case studies from India. Asian Journal of Environment & Ecology 19, 142–150.

[brv13155-bib-0118] Kotila, M. , Suominen, K. M. , Vasko, V. V. , Blomberg, A. S. , Lehikoinen, A. , Andersson, T. , Aspi, J. , Cederberg, T. , Hänninen, J. , Inkinen, J. , Koskinen, J. , Lundberg, G. , Mäkinen, K. , Rontti, M. , Snickars, M. , *et al*. (2023). Large‐scale long‐term passive‐acoustic monitoring reveals spatio‐temporal activity patterns of boreal bats. Ecography 2023, e06617.

[brv13155-bib-0119] Kowarski, K. A. & Moors‐Murphy, H. (2021). A review of big data analysis methods for baleen whale passive acoustic monitoring. Marine Mammal Science 37, 652–673.

[brv13155-bib-0120] Krause, B. L. (1993). The niche hypothesis: a virtual symphony of animal sounds, the origins of musical expression and the health of habitats. The Soundscape Newsletter 6, 6–10.

[brv13155-bib-0121] Laiolo, P. , Rolando, A. , Delestrade, A. & Sanctis, A. d. (2001). Geographical variation in the calls of the choughs. The Condor 103, 287–297.

[brv13155-bib-0122] Laurijs, K. A. , Briefer, E. F. , Reimert, I. & Webb, L. E. (2021). Vocalisations in farm animals: a step towards positive welfare assessment. Applied Animal Behaviour Science 236, 105264.

[brv13155-bib-0123] Law, B. , Gonsalves, L. , Burgar, J. , Brassil, T. , Kerr, I. , Wilmott, L. , Madden, K. , Smith, M. , Mella, V. , Crowther, M. , Krockenberger, M. , Rus, A. , Pietsch, R. , Truskinger, A. , Eichinski, P. , *et al*. (2021). Estimating and validating koala. Wildlife Research 49, 438–448.

[brv13155-bib-0124] LeCun, Y. , Bengio, Y. & Hinton, G. (2015). Deep learning. Nature 521, 436–444. 26017442 10.1038/nature14539

[brv13155-bib-0125] Leighton, G. M. & Birmingham, T. (2021). Multiple factors affect the evolution of repertoire size across birds. Behavioral Ecology 32, 380–385.

[brv13155-bib-0126] Leroux, M. , Al‐Khudhairy, O. G. , Perony, N. & Townsend, S. W. (2021). Chimpanzee voice prints? Insights from transfer learning experiments from human voices. arXiv. http://arxiv.org/abs/2112.08165. Accessed 6 July 2023.

[brv13155-bib-0127] Lever, J. , Krzywinski, M. & Altman, N. (2016). Classification evaluation. Nature Methods 13, 603–604.

[brv13155-bib-0128] Li, N. , Ren, Z. , Li, D. & Zeng, L. (2020). Automated techniques for monitoring the behaviour and welfare of broilers and laying hens: towards the goal of precision livestock farming. Animal 14, 617–625. 31566170 10.1017/S1751731119002155

[brv13155-bib-0129] Liebal, K. & Oña, L. (2018). Different approaches to meaning in primate gestural and vocal communication. Frontiers in Psychology 9, 478. 29692748 10.3389/fpsyg.2018.00478 PMC5902706

[brv13155-bib-0130] Lin, T. , Wang, Y. , Liu, X. & Qiu, X. (2022). A survey of transformers. AI Open 3, 111–132.

[brv13155-bib-0131] Long, R. A. , MacKay, P. , Ray, J. & Zielinski, W. (2012). Noninvasive Survey Methods for Carnivores. Island Press, Washington, D.C.

[brv13155-bib-0132] Lostanlen, V. , Salamon, J. , Farnsworth, A. , Kelling, S. & Bello, J. P. (2018). Birdvox‐full‐night: a dataset and benchmark for avian flight call detection. In *2018 IEEE International Conference on Acoustics, Speech and Signal Processing (ICASSP), IEEE, Calgary, AB, Canada*.

[brv13155-bib-0133] Maaten, L. v. d. & Hinton, G. (2008). Visualizing data using t‐SNE. Journal of Machine Learning Research 9, 2579–2605.

[brv13155-bib-0134] MacKenzie, D. I. , Nichols, J. D. , Hines, J. E. , Knutson, M. G. & Franklin, A. B. (2003). Estimating site occupancy, colonization, and local extinction when a species is detected imperfectly. Ecology 84, 2200–2207.

[brv13155-bib-0135] Manser, M. B. , Jansen, D. A. W. A. M. , Graw, B. , Hollén, L. I. , Bousquet, C. A. H. , Furrer, R. D. & le Roux, A. (2014). Chapter six ‐ vocal complexity in Meerkats and other mongoose species. In Advances in the Study of Behavior (eds M. Naguib , L. Barrett , H. J. Brockmann , S. Healy , J. C. Mitani , T. J. Roper and L. W. Simmons ), pp. 281–310. Academic Press, Cambridge.

[brv13155-bib-0136] Manteuffel, G. , Puppe, B. & Schön, P. C. (2004). Vocalization of farm animals as a measure of welfare. Applied Animal Behaviour Science 88, 163–182.

[brv13155-bib-0137] Mao, A. , Giraudet, C. S. E. , Liu, K. , De Almeida Nolasco, I. , Xie, Z. , Xie, Z. , Gao, Y. , Theobald, J. , Bhatta, D. , Stewart, R. & McElligott, A. G. (2022). Automated identification of chicken distress vocalizations using deep learning models. Journal of the Royal Society Interface 19, 20210921. 35765806 10.1098/rsif.2021.0921 PMC9240672

[brv13155-bib-0138] Marin‐Cudraz, T. , Muffat‐Joly, B. , Novoa, C. , Aubry, P. , Desmet, J.‐F. , Mahamoud‐Issa, M. , Nicolè, F. , Van Niekerk, M. H. , Mathevon, N. & Sèbe, F. (2019). Acoustic monitoring of rock ptarmigan: a multi‐year comparison with point‐count protocol. Ecological Indicators 101, 710–719.

[brv13155-bib-0139] Marques, T. A. , Thomas, L. , Martin, S. W. , Mellinger, D. K. , Ward, J. A. , Moretti, D. J. , Harris, D. & Tyack, P. L. (2013). Estimating animal population density using passive acoustics. Biological Reviews 88, 287–309. 23190144 10.1111/brv.12001 PMC3743169

[brv13155-bib-0140] Marques, T. A. , Thomas, L. , Ward, J. , DiMarzio, N. & Tyack, P. L. (2009). Estimating cetacean population density using fixed passive acoustic sensors: an example with Blainville's beaked whales. The Journal of the Acoustical Society of America 125, 1982–1994. 19354374 10.1121/1.3089590

[brv13155-bib-0141] Martin, K. , Adam, O. , Obin, N. & Dufour, V. (2022). Rookognise: acoustic detection and identification of individual rooks in field recordings using multi‐task neural networks. Ecological Informatics 72, 101818.

[brv13155-bib-0142] McComb, K. & Semple, S. (2005). Coevolution of vocal communication and sociality in primates. Biology Letters 1, 381–385. 17148212 10.1098/rsbl.2005.0366 PMC1626386

[brv13155-bib-0143] McDonald, M. , Hildebrand, J. & Mesnick, S. (2009). Worldwide decline in tonal frequencies of blue whale songs. Endangered Species Research 9, 13–21.

[brv13155-bib-0144] McDonald, M. A. & Fox, C. G. (1999). Passive acoustic methods applied to fin whale population density estimation. The Journal of the Acoustical Society of America 105, 2643–2651.

[brv13155-bib-0145] McInnes, L. , Healy, J. & Melville, J. (2020). UMAP: uniform manifold approximation and projection for dimension reduction. arXiv. http://arxiv.org/abs/1802.03426. Accessed 7 August 2023.

[brv13155-bib-0146] Mcloughlin, M. P. , Stewart, R. & McElligott, A. G. (2019). Automated bioacoustics: methods in ecology and conservation and their potential for animal welfare monitoring. Journal of the Royal Society Interface 16, 20190225. 31213168 10.1098/rsif.2019.0225 PMC6597774

[brv13155-bib-0147] Mellinger, D. K. & Clark, C. W. (1997). Methods for automatic detection of mysticete sounds. Marine and Freshwater Behaviour and Physiology 29, 163–181.

[brv13155-bib-0148] Mennill, D. J. , Battiston, M. , Wilson, D. R. , Foote, J. R. & Doucet, S. M. (2012). Field test of an affordable, portable, wireless microphone array for spatial monitoring of animal ecology and behaviour. Methods in Ecology and Evolution 3, 704–712.

[brv13155-bib-0149] Metcalf, O. , Abrahams, C. , Ashington, B. , Baker, E. , Bradfer‐Lawrence, T. , Browning, E. , Carruthers‐Jones, J. , Darby, J. , Dick, J. , Eldridge, A. , Elliot, D. , Heath, B. , Howden‐Leach, P. , Johston, A. , Lees, A. , *et al*. (2023). Good Practice Guidelines for Long‐Term Ecoacoustic Monitoring in the UK. The UK Acoustics Network.

[brv13155-bib-0150] Meyer, D. , Hodges, J. K. , Rinaldi, D. , Wijaya, A. , Roos, C. & Hammerschmidt, K. (2012). Acoustic structure of male loud‐calls support molecular phylogeny of Sumatran and Javanese leaf monkeys (genus *Presbytis*). BMC Evolutionary Biology 12, 16. 22305415 10.1186/1471-2148-12-16 PMC3295661

[brv13155-bib-0151] Miller, B. S. , Madhusudhana, S. , Aulich, M. G. & Kelly, N. (2023). Deep learning algorithm outperforms experienced human observer at detection of blue whale D‐calls: a double‐observer analysis. Remote Sensing in Ecology and Conservation 9, 104–116.

[brv13155-bib-0152] Ministry of Local Government and Modernisation (2021). Norwegian data centres ‐ sustainable, digital powerhouses. Plan, regjeringen.no. Government.no. https://www.regjeringen.no/en/dokumenter/norwegian-data-centres-sustainable-digital-powerhouses/id2867155/. Accessed 7 August 2023.

[brv13155-bib-0153] Mitrovic, D. , Zeppelzauer, M. & Breiteneder, C. (2006). Discrimination and retrieval of animal sounds. *In* 2006 12th International Multi‐Media Modelling Conference, IEEE, Beijing, China.

[brv13155-bib-0154] Monczak, A. , Berry, A. , Kehrer, C. & Montie, E. W. (2017). Long‐term acoustic monitoring of fish calling provides baseline estimates of reproductive timelines in the May River estuary, southeastern USA. Marine Ecology Progress Series 581, 1–19.

[brv13155-bib-0155] Morrison, A. & Novikova, A. (2023). Monitoring technologies for animal welfare: a review of aspirations and deployments in zoos. In Proceedings of the Future Technologies Conference (FTC) 2022, Volume 3 (ed. K. Arai ), pp. 155–178. Springer International Publishing, Cham.

[brv13155-bib-0156] Mutanu, L. , Gohil, J. , Gupta, K. , Wagio, P. & Kotonya, G. (2022). A review of automated bioacoustics and general acoustics classification research. Sensors 22, 8361. 36366061 10.3390/s22218361 PMC9658612

[brv13155-bib-0157] Narasimhan, R. , Fern, X. Z. & Raich, R. (2017). Simultaneous segmentation and classification of bird song using CNN. In *2017 IEEE International Conference on Acoustics, Speech and Signal Processing (ICASSP)*, pp. 146–150. IEEE, New Orleans, LA, USA.

[brv13155-bib-0158] Nelson, D. A. (2000). Song overproduction, selective attrition and song dialects in the white‐crowned sparrow. Animal Behaviour 60, 887–898. 11124888 10.1006/anbe.2000.1560

[brv13155-bib-0159] Nicholson, D. (2023). Crowsetta: a python tool to work with any format for annotating animal vocalizations and bioacoustics data. Journal of Open Source Software 8, 5338.

[brv13155-bib-0160] Nijman, V. (2007). Effects of vocal behaviour on abundance estimates of rainforest galliforms. Acta Ornithologica 42, 186–190.

[brv13155-bib-0161] Nolasco, I. , Singh, S. , Morfi, V. , Lostanlen, V. , Strandburg‐Peshkin, A. , Vidaña‐Vila, E. , Gill, L. , Pamuła, H. , Whitehead, H. , Kiskin, I. , Jensen, F. H. , Morford, J. , Emmerson, M. G. , Versace, E. , Grout, E. , *et al*. (2023). Learning to detect an animal sound from five examples. Ecological Informatics 77, 102258.

[brv13155-bib-0162] Obrist, M. K. , Pavan, G. , Sueur, J. , Riede, K. , Llusia, D. & Márquez, R. (2010). Bioacoustics approaches in biodiversity inventories. ABC Taxa 8, 68–99.

[brv13155-bib-0163] Odom, K. J. , Araya‐Salas, M. , Morano, J. L. , Ligon, R. A. , Leighton, G. M. , Taff, C. C. , Dalziell, A. H. , Billings, A. C. , Germain, R. R. , Pardo, M. , de Andrade, L. G. , Hedwig, D. , Keen, S. C. , Shiu, Y. , Charif, R. A. , *et al*. (2021). Comparative bioacoustics: a roadmap for quantifying and comparing animal sounds across diverse taxa. Biological Reviews 96, 1135–1159. 33652499 10.1111/brv.12695

[brv13155-bib-0164] Ogutu, J. O. , Piepho, H.‐P. , Said, M. Y. , Ojwang, G. O. , Njino, L. W. , Kifugo, S. C. & Wargute, P. W. (2016). Extreme wildlife declines and concurrent increase in livestock numbers in Kenya: what are the causes? PLoS One 11, e0163249. 27676077 10.1371/journal.pone.0163249 PMC5039022

[brv13155-bib-0165] Oliver, R. Y. , Ellis, D. P. W. , Chmura, H. E. , Krause, J. S. , Pérez, J. H. , Sweet, S. K. , Gough, L. , Wingfield, J. C. & Boelman, N. T. (2018). Eavesdropping on the Arctic: automated bioacoustics reveal dynamics in songbird breeding phenology. Science Advances 4, eaaq1084. 29938220 10.1126/sciadv.aaq1084 PMC6010323

[brv13155-bib-0166] Oswald, J. N. , Erbe, C. , Gannon, W. L. , Madhusudhana, S. & Thomas, J. A. (2022). Detection and classification methods for animal sounds. In Exploring Animal Behavior through Sound: Volume 1: Methods (eds C. Erbe and J. A. Thomas ), pp. 269–317. Springer International Publishing, Cham.

[brv13155-bib-0167] Parrilla, A. G. A. & Stowell, D. (2022). Polyphonic sound event detection for highly dense birdsong scenes. arXiv. http://arxiv.org/abs/2207.06349. Accessed 13 June 2024.

[brv13155-bib-0168] Pérez‐Granados, C. & Traba, J. (2021). Estimating bird density using passive acoustic monitoring: a review of methods and suggestions for further research. Ibis 163, 765–783.

[brv13155-bib-0169] Petso, T. , Jamisola, R. S. & Mpoeleng, D. (2021). Review on methods used for wildlife species and individual identification. European Journal of Wildlife Research 68, 3.

[brv13155-bib-0170] Politis, A. , Mesaros, A. , Adavanne, S. , Heittola, T. & Virtanen, T. (2021). Overview and evaluation of sound event localization and detection in DCASE 2019. IEEE/ACM Transactions on Audio, Speech, and Language Processing 29, 684–698.

[brv13155-bib-0171] Powell, R. (2000). Animal home ranges and territories and home range estimators. In Research Techniques in Animal Ecology: Controversies and Consequences, pp. 65–110. Colombia: Colombia University Press.

[brv13155-bib-0172] Qian, T. , Deng, G. , Li, Y. & Yang, D. (2023). Description of the advertisement call of *Boulenophrys nanlingensis* (Anura, Megophryidae), with a case of individual identification using its dorsum pattern. Herpetozoa 36, 123–128.

[brv13155-bib-0173] Rice, A. , Širović, A. , Trickey, J. S. , Debich, A. J. , Gottlieb, R. S. , Wiggins, S. M. , Hildebrand, J. A. & Baumann‐Pickering, S. (2021). Cetacean occurrence in the Gulf of Alaska from long‐term passive acoustic monitoring. Marine Biology 168, 72.

[brv13155-bib-0174] Riesch, R. , Barrett‐Lennard, L. G. , Ellis, G. M. , Ford, J. K. B. & Deecke, V. B. (2012). Cultural traditions and the evolution of reproductive isolation: ecological speciation in killer whales? Biological Journal of the Linnean Society 106, 1–17.

[brv13155-bib-0175] Romero‐Mujalli, D. , Bergmann, T. , Zimmermann, A. & Scheumann, M. (2021). Utilizing DeepSqueak for automatic detection and classification of mammalian vocalizations: a case study on primate vocalizations. Scientific Reports 11, 24463. 34961788 10.1038/s41598-021-03941-1 PMC8712519

[brv13155-bib-0176] Ross, S. R. P.‐J. , Friedman, N. R. , Dudley, K. L. , Yoshimura, M. , Yoshida, T. & Economo, E. P. (2018). Listening to ecosystems: data‐rich acoustic monitoring through landscape‐scale sensor networks. Ecological Research 33, 135–147.

[brv13155-bib-0177] Ross, S. R. P.‐J. , O'Connell, D. P. , Deichmann, J. L. , Desjonquères, C. , Gasc, A. , Phillips, J. N. , Sethi, S. S. , Wood, C. M. & Burivalova, Z. (2023). Passive acoustic monitoring provides a fresh perspective on fundamental ecological questions. Functional Ecology 37, 959–975.

[brv13155-bib-0178] Rothstein, S. I. & Fleischer, R. C. (1987). Vocal dialects and their possible relation to honest status signalling in the brown‐headed cowbird. The Condor 89, 1–23.

[brv13155-bib-0179] Saeed, A. , Grangier, D. & Zeghidour, N. (2021). Contrastive learning of general‐purpose audio representations. In *ICASSP 2021–2021 IEEE International Conference on Acoustics, Speech and Signal Processing (ICASSP)*, pp. 3875–3879.

[brv13155-bib-0180] Sainburg, T. , Thielk, M. & Gentner, T. Q. (2020). Latent space visualization, characterization, and generation of diverse vocal communication signals. bioRxiv. 10.1101/870311v2. Accessed 13 June 2024.

[brv13155-bib-0181] Sandbrook, C. , Clark, D. , Toivonen, T. , Simlai, T. , O'Donnell, S. , Cobbe, J. & Adams, W. (2021). Principles for the socially responsible use of conservation monitoring technology and data. Conservation Science and Practice 3, e374.

[brv13155-bib-0182] Sarkar, E. & Magimai Doss, M. (eds) (2023). Can self‐supervised neural representations pre‐trained on human speech distinguish animal callers? In Interspeech Conference of the International Speech Communication Association 2023. ISCA, Dublin, Ireland.

[brv13155-bib-0183] Sattar, F. (2023). A new acoustical autonomous method for identifying endangered whale calls: a case study of blue whale and fin whale. Sensors 23, 3048. 36991759 10.3390/s23063048 PMC10056851

[brv13155-bib-0184] Schultze, S. , Gruenefeld, U. & Boll, S. (2020). Demystifying deep learning: developing a learning app for beginners to gain practical experience. *In Proceedings of the Mensch und Computer 2020 Workshop*.

[brv13155-bib-0185] Sebastián‐González, E. , Pang‐Ching, J. , Barbosa, J. M. & Hart, P. (2015). Bioacoustics for species management: two case studies with a Hawaiian forest bird. Ecology and Evolution 5, 4696–4705. 26668733 10.1002/ece3.1743 PMC4670053

[brv13155-bib-0186] Sethi, S. S. , Jones, N. S. , Fulcher, B. D. , Picinali, L. , Clink, D. J. , Klinck, H. , Orme, C. D. L. , Wrege, P. H. & Ewers, R. M. (2020). Characterizing soundscapes across diverse ecosystems using a universal acoustic feature set. Proceedings of the National Academy of Sciences 117, 17049–17055. 10.1073/pnas.2004702117 PMC7382238 32636258

[brv13155-bib-0187] Sharma, K. , Fiechter, M. , George, T. , Young, J. , Alexander, J. S. , Bijoor, A. , Suryawanshi, K. & Mishra, C. (2020). Conservation and people: towards an ethical code of conduct for the use of camera traps in wildlife research. Ecological Solutions and Evidence 1, e12033.

[brv13155-bib-0188] Sharma, S. , Sato, K. & Gautam, B. P. (2022). Bioacoustics monitoring of wildlife using artificial intelligence: a methodological literature review. In *2022 International Conference on Networking and Network Applications (NaNA)*, pp. 1–9.

[brv13155-bib-0189] Sharpe, F. , Bolton, M. , Sheldon, R. & Ratcliffe, N. (2009). Effects of color banding, radio tagging, and repeated handling on the condition and survival of Lapwing chicks and consequences for estimates of breeding productivity. Journal of Field Ornithology 80, 101–110.

[brv13155-bib-0190] Shiu, Y. , Palmer, K. J. , Roch, M. A. , Fleishman, E. , Liu, X. , Nosal, E.‐M. , Helble, T. , Cholewiak, D. , Gillespie, D. & Klinck, H. (2020). Deep neural networks for automated detection of marine mammal species. Scientific Reports 10, 607. 31953462 10.1038/s41598-020-57549-y PMC6969184

[brv13155-bib-0191] Slocombe, K. E. , Waller, B. M. & Liebal, K. (2011). The language void: the need for multimodality in primate communication research. Animal Behaviour 81, 919–924.

[brv13155-bib-0192] Smith, B. R. , Root‐Gutteridge, H. , Butkiewicz, H. , Dassow, A. , Fontaine, A. C. , Markham, A. , Owens, J. , Schindler, L. , Wijers, M. & Kershenbaum, A. (2021). Acoustic localisation of wildlife with low‐cost equipment: lower sensitivity, but no loss of precision. Wildlife Research 49, 372–381.

[brv13155-bib-0193] Soulsbury, C. , Gray, H. , Smith, L. , Braithwaite, V. , Cotter, S. , Elwood, R. W. , Wilkinson, A. & Collins, L. M. (2020). The welfare and ethics of research involving wild animals: a primer. Methods in Ecology and Evolution 11, 1164–1181.

[brv13155-bib-0194] Spillmann, B. , Schaik, C. P. v. , Setia, T. M. & Sadjadi, S. O. (2017). Who shall I say is calling? Validation of a caller recognition procedure in Bornean flanged male orangutan (*Pongo pygmaeus wurmbii*) long calls. Bioacoustics 26, 109–120.

[brv13155-bib-0195] Staaterman, E. , Ogburn, M. B. , Altieri, A. H. , Brandl, S. J. , Whippo, R. , Seemann, J. , Goodison, M. & Duffy, J. E. (2017). Bioacoustic measurements complement visual biodiversity surveys: preliminary evidence from four shallow marine habitats. Marine Ecology Progress Series 575, 207–215.

[brv13155-bib-0196] Stowell, D. (2022). Computational bioacoustics with deep learning: a review and roadmap. PeerJ 10, e13152. 35341043 10.7717/peerj.13152 PMC8944344

[brv13155-bib-0197] Stowell, D. , Wood, M. D. , Pamuła, H. , Stylianou, Y. & Glotin, H. (2019). Automatic acoustic detection of birds through deep learning: the first bird audio detection challenge. Methods in Ecology and Evolution 10, 368–380.

[brv13155-bib-0198] Sueur, J. , Farina, A. , Gasc, A. , Pieretti, N. & Pavoine, S. (2014). Acoustic indices for biodiversity assessment and landscape investigation. Acta Acustica united with Acustica 100, 772–781.

[brv13155-bib-0199] Sugai, L. S. M. & Llusia, D. (2019). Bioacoustic time capsules: using acoustic monitoring to document biodiversity. Ecological Indicators 99, 149–152.

[brv13155-bib-0200] Sugai, L. S. M. , Silva, T. S. F. , Ribeiro, J. W. Jr. & Llusia, D. (2019). Terrestrial passive acoustic monitoring: review and perspectives. Bioscience 69, 15–25.

[brv13155-bib-0201] Teixeira, D. , Maron, M. & van Rensburg, B. J. (2019). Bioacoustic monitoring of animal vocal behavior for conservation. Conservation Science and Practice 1, e72.

[brv13155-bib-0202] Torti, V. , Valente, D. , Gregorio, C. D. , Comazzi, C. , Miaretsoa, L. , Ratsimbazafy, J. , Giacoma, C. & Gamba, M. (2018). Call and be counted! Can we reliably estimate the number of callers in the indri's (*Indri indri*) song? PLoS One 13, e0201664. 30075022 10.1371/journal.pone.0201664 PMC6075759

[brv13155-bib-0203] Towsey, M. , Planitz, B. , Nantes, A. , Wimmer, J. & Roe, P. (2012). A toolbox for animal call recognition. Bioacoustics 21, 107–125.

[brv13155-bib-0204] Turian, J. , Shier, J. , Khan, H. R. , Raj, B. , Schuller, B. W. , Steinmetz, C. J. , Malloy, C. , Tzanetakis, G. , Velarde, G. , McNally, K. , Henry, M. , Pinto, N. , Noufi, C. , Clough, C. , Herremans, D. , *et al*. (2022). HEAR: holistic evaluation of audio representations. In *Proceedings of the NeurIPS 2021 Competitions and Demonstrations Track*, pp. 125–145.

[brv13155-bib-0205] Uetz, G. W. & Roberts, J. A. (2002). Multisensory cues and multimodal communication in spiders: insights from video/audio playback studies. Brain Behavior and Evolution 59, 222–230. 12138342 10.1159/000064909

[brv13155-bib-0206] Usman, A. M. , Ogundile, O. O. & Versfeld, D. J. J. (2020). Review of automatic detection and classification techniques for cetacean vocalization. IEEE Access 8, 105181–105206.

[brv13155-bib-0207] Van Segbroeck, M. , Knoll, A. T. , Levitt, P. & Narayanan, S. (2017). MUPET—Mouse Ultrasonic Profile ExTraction: a signal processing tool for rapid and unsupervised analysis of ultrasonic vocalizations. Neuron 94, 465–485. 28472651 10.1016/j.neuron.2017.04.005 PMC5939957

[brv13155-bib-0208] Velásquez, N. A. , Marambio, J. , Brunetti, E. , Méndez, M. A. , Vásquez, R. A. & Penna, M. (2013). Bioacoustic and genetic divergence in a frog with a wide geographical distribution. Biological Journal of the Linnean Society 110, 142–155.

[brv13155-bib-0209] Venkatesh, S. , Moffat, D. & Miranda, E. R. (2022). You only hear once: a YOLO‐like algorithm for audio segmentation and sound event detection. Applied Sciences 12, 3293.

[brv13155-bib-0210] Vickers, W. , Milner, B. , Risch, D. & Lee, R. (2021). Robust North Atlantic right whale detection using deep learning models for denoising. The Journal of the Acoustical Society of America 149, 3797–3812. 34241455 10.1121/10.0005128

[brv13155-bib-0211] Volodina, E. V. & Volodin, I. A. (1999). Bioacoustics in zoos: a review of applications and perspectives. International Zoo News 46, 208–213.

[brv13155-bib-0212] Vu, T. T. , Nguyen, T. C. , Doherty, P. F. Jr. , Nguyen, H. T. , Clink, D. J. , Nguyen, M. D. , Dong, H. T. & Giang, T. T. (2023). Using mobile smartphones and bioacoustics to monitor endangered bird species. Ibis 165, 1368–1377.

[brv13155-bib-0213] Vu, T. T. & Tran, L. M. (2019). An application of autonomous recorders for gibbon monitoring. International Journal of Primatology 40, 169–186.

[brv13155-bib-0214] Wang, Z. , She, Q. & Ward, T. E. (2021). Generative adversarial networks in computer vision: a survey and taxonomy. ACM Computing Surveys 54(37), 1–37.

[brv13155-bib-0215] Ward, J. , Fitzpatrick, M. , DiMarzio, N. , Moretti, D. & Morrissey, R. (2000). New algorithms for open ocean marine mammal monitoring. In *OCEANS 2000 MTS/IEEE Conference and Exhibition. Conference Proceedings (Cat. No.00CH37158)* pp. 1749–1752, vol. 3. IEEE, Providence, RI, USA.

[brv13155-bib-0216] Wijers, M. , Loveridge, A. , Macdonald, D. W. & Markham, A. (2021). CARACAL: a versatile passive acoustic monitoring tool for wildlife research and conservation. Bioacoustics 30, 41–57.

[brv13155-bib-0217] Wildlife Acoustics, Inc (2024). Kaleidoscope pro analysis software. Wildlife Acoustics. https://www.wildlifeacoustics.com/products/kaleidoscope‐pro. Accessed 13 June 2024.

[brv13155-bib-0218] Wilkinson, M. D. , Dumontier, M. , Aalbersberg, I. J. , Appleton, G. , Axton, M. , Baak, A. , Blomberg, N. , Boiten, J.‐W. , da Silva Santos, L. B. , Bourne, P. E. , Bouwman, J. , Brookes, A. J. , Clark, T. , Crosas, M. , Dillo, I. , *et al*. (2016). The FAIR guiding principles for scientific data management and stewardship. Scientific Data 3, 160018. 26978244 10.1038/sdata.2016.18 PMC4792175

[brv13155-bib-0219] Willacy, R. J. , Mahony, M. & Newell, D. A. (2015). If a frog calls in the forest: bioacoustic monitoring reveals the breeding phenology of the endangered Richmond Range mountain frog (*Philoria richmondensis*). Austral Ecology 40, 625–633.

[brv13155-bib-0220] Wisdom, S. , Tzinis, E. , Erdogan, H. , Weiss, R. , Wilson, K. & Hershey, J. (2020). Unsupervised sound separation using mixture invariant training. In Advances in Neural Information Processing Systems 36 (NeurIPS 2023), pp. 3846–3857.

[brv13155-bib-0221] Wolf, C. & Ripple, W. J. (2016). Prey depletion as a threat to the world's large carnivores. Royal Society Open Science 3, 160252. 27853599 10.1098/rsos.160252 PMC5108949

[brv13155-bib-0222] Wood, C. M. & Peery, M. Z. (2022). What does “occupancy” mean in passive acoustic surveys? Ibis 164, 1295–1300.

[brv13155-bib-0223] Wu, S.‐H. , Chang, H.‐W. , Lin, R.‐S. & Tuanmu, M.‐N. (2022). SILIC: a cross database framework for automatically extracting robust biodiversity information from soundscape recordings based on object detection and a tiny training dataset. Ecological Informatics 68, 101534.

[brv13155-bib-0224] Xie, J. , Colonna, J. G. & Zhang, J. (2021). Bioacoustic signal denoising: a review. Artificial Intelligence Review 54, 3575–3597.

[brv13155-bib-0225] Xie, J. , Zhong, Y. , Zhang, J. , Liu, S. , Ding, C. & Triantafyllopoulos, A. (2023). A review of automatic recognition technology for bird vocalizations in the deep learning era. Ecological Informatics 73, 101927.

[brv13155-bib-0226] Yang, W. , Chang, W. , Song, Z. , Zhang, Y. & Wang, X. (2021). Transfer learning for denoising the echolocation clicks of finless porpoise (*Neophocaena phocaenoides sunameri*) using deep convolutional autoencoders. The Journal of the Acoustical Society of America 150, 1243–1250. 34470267 10.1121/10.0005887

[brv13155-bib-0227] Yin, S. , Liu, C. , Zhang, Z. , Lin, Y. , Wang, D. , Tejedor, J. , Zheng, T. F. & Li, Y. (2015). Noisy training for deep neural networks in speech recognition. EURASIP Journal on Audio, Speech, and Music Processing 2015, 2.

[brv13155-bib-0228] Yu, Y. , Si, X. , Hu, C. & Zhang, J. (2019). A review of recurrent neural networks: LSTM cells and network architectures. Neural Computation 31, 1235–1270. 31113301 10.1162/neco_a_01199

[brv13155-bib-0229] Zhang, H. , Cisse, M. , Dauphin, Y. N. & Lopez‐Paz, D. (2018). Mixup: beyond empirical risk minimization. arXiv. http://arxiv.org/abs/1710.09412. Accessed 13 June 2024.

[brv13155-bib-0230] Zhang, Z. , Zhang, H. , He, Y. & Liu, T. (2022). A review in the automatic detection of pigs behavior with sensors. Journal of Sensors 2022, 4519539.

[brv13155-bib-0231] Zhuang, F. , Qi, Z. , Duan, K. , Xi, D. , Zhu, Y. , Zhu, H. , Xiong, H. & He, Q. (2021). A comprehensive survey on transfer learning. Proceedings of the IEEE 109, 43–76.

[brv13155-bib-0232] Zimmer, W. M. X. (2011). Passive Acoustic Monitoring of Cetaceans. Cambridge University Press, Cambridge, UK.

[brv13155-bib-0233] Zwerts, J. A. , Stephenson, P. J. , Maisels, F. , Rowcliffe, M. , Astaras, C. , Jansen, P. A. , van der Waarde, J. , Sterck, L. E. H. M. , Verweij, P. A. , Bruce, T. , Brittain, S. & van Kuijk, M. (2021). Methods for wildlife monitoring in tropical forests: comparing human observations, camera traps, and passive acoustic sensors. Conservation Science and Practice 3, e568.

